# Birth and death notification via mobile devices: a mixed methods systematic review

**DOI:** 10.1002/14651858.CD012909.pub2

**Published:** 2021-07-16

**Authors:** Lavanya Vasudevan, Claire Glenton, Nicholas Henschke, Nicola Maayan, John Eyers, Marita S Fønhus, Tigest Tamrat, Garrett L Mehl, Simon Lewin

**Affiliations:** Center for Health Policy and Inequalities ResearchDuke Global Health InstituteDurhamNorth CarolinaUSA; Department of Family Medicine and Community HealthDuke UniversityDurhamNorth CarolinaUSA; Norwegian Institute of Public HealthOsloNorway; Cochrane ResponseCochraneLondonUK; Independent consultantLondonUK; North CurryUK; Department of Sexual and Reproductive HealthWorld Health OrganizationGenevaSwitzerland; Health Systems Research UnitSouth African Medical Research CouncilCape TownSouth Africa

**Keywords:** Humans, Bias, Birth Certificates, Computers, Handheld, Controlled Before-After Studies, Death Certificates, Health Services Accessibility, Rural Population, Time Factors

## Abstract

**Background:**

Ministries of health, donors, and other decision‐makers are exploring how they can use mobile technologies to acquire accurate and timely statistics on births and deaths. These stakeholders have called for evidence‐based guidance on this topic. This review was carried out to support World Health Organization (WHO) recommendations on digital interventions for health system strengthening.

**Objectives:**

*Primary objective:* To assess the effects of birth notification and death notification via a mobile device, compared to standard practice.

*Secondary objectives:* To describe the range of strategies used to implement birth and death notification via mobile devices and identify factors influencing the implementation of birth and death notification via mobile devices.

**Search methods:**

We searched CENTRAL, MEDLINE, Embase, the Global Health Library, and POPLINE (August 2, 2019). We searched two trial registries (August 2, 2019). We also searched Epistemonikos for related systematic reviews and potentially eligible primary studies (August 27, 2019). We conducted a grey literature search using mHealthevidence.org (August 15, 2017) and issued a call for papers through popular digital health communities of practice. Finally, we conducted citation searches of included studies in Web of Science and Google Scholar (May 15, 2020). We searched for studies published after 2000 in any language.

**Selection criteria:**

For the *primary objective,* we included individual and cluster‐randomised trials; cross‐over and stepped‐wedge study designs; controlled before‐after studies, provided they have at least two intervention sites and two control sites; and interrupted time series studies. For the *secondary objectives*, we included any study design, either quantitative, qualitative, or descriptive, that aimed to describe current strategies for birth and death notification via mobile devices; or to explore factors that influence the implementation of these strategies, including studies of acceptability or feasibility.

For the *primary objective*, we included studies that compared birth and death notification via mobile devices with standard practice. For the *secondary objectives,* we included studies of birth and death notification via mobile device as long as we could extract data relevant to our secondary objectives.

We included studies of all cadres of healthcare providers, including lay health workers; administrative, managerial, and supervisory staff; focal individuals at the village or community level; children whose births were being notified and their parents/caregivers; and individuals whose deaths were being notified and their relatives/caregivers.

**Data collection and analysis:**

For the *primary objective,* two authors independently screened all records, extracted data from the included studies and assessed risk of bias. For the analyses of the primary objective, we reported means and proportions, where appropriate. We used the Grading of Recommendations Assessment, Development and Evaluation (GRADE) approach to assess the certainty of the evidence and we prepared a 'Summary of Findings' table.

For the *secondary objectives,* two authors screened all records, one author extracted data from the included studies and assessed methodological limitations using the WEIRD tool and a second author checked the data and assessments. We carried out a framework analysis using the Supporting the Use of Research Evidence (SURE) framework to identify themes in the data. We used the GRADE‐CERQual (Confidence in the Evidence from Reviews of Qualitative research) approach to assess our confidence in the evidence and we prepared a 'Summary of Qualitative Findings' table.

**Main results:**

For the *primary objective*, we included one study, which used a controlled before‐after study design. The study was conducted in Lao People’s Democratic Republic and assessed the effect of using mobile devices for birth notification on outcomes related to coverage and timeliness of Hepatitis B vaccination. However, we are uncertain of the effect of this approach on these outcomes because the certainty of this evidence was assessed as very low. The included study did not assess resource use or unintended consequences. For the primary objective, we did not identify any studies using mobile devices for death notification.

For the *secondary objective*, we included 21 studies. All studies were conducted in low‐ or middle‐income settings. They focussed on identification of births and deaths in rural, remote, or marginalised populations who are typically under‐represented in civil registration processes or traditionally seen as having poor access to health services.

The review identified several factors that could influence the implementation of birth‐death notification via mobile device. These factors were tied to the health system, the person responsible for notifying, the community and families; and include:

‐ Geographic barriers that could prevent people’s access to birth‐death notification and post‐notification services

‐ Access to health workers and other notifiers with enough training, supervision, support, and incentives

‐ Monitoring systems that ensure the quality and timeliness of the birth and death data

‐ Legal frameworks that allow births and deaths to be notified by mobile device and by different types of notifiers

‐ Community awareness of the need to register births and deaths

‐ Socio‐cultural norms around birth and death

‐ Government commitment

‐ Cost to the system, to health workers and to families

‐ Access to electricity and network connectivity, and compatibility with existing systems

‐ Systems that protect data confidentiality

We have low to moderate confidence in these findings. This was mainly because of concerns about methodological limitations and data adequacy.

**Authors' conclusions:**

We need more, well‐designed studies of the effect of birth and death notification via mobile devices and on factors that may influence its implementation.

## Plain language summary

**Birth and death notification via mobile devices: a mixed methods review**

**What is the aim of this review?**

In this Cochrane Review, we aimed to assess the effect of using mobile devices to notify births and deaths. We also aimed to describe how these mobile solutions are being used in practice and the factors that influence their use. We collected and analysed all relevant studies to answer these questions.

**Key messages**

We know very little about the effects of using mobile devices to notify births and deaths. Factors that can influence the implementation of this approach include factors tied to the health system and the notification system, the person responsible for notifying, the community, and the families involved.

**What was studied in the review?**

By registering notified births and deaths, governments can track the health of their population, identify needs and problems, and design better services. In many countries, births and deaths are not properly registered. Sometimes this is because government systems are poorly designed to facilitate registration, government workers do not have proper training, people live far from government services, or are not aware of the need to register births or deaths. In many cases, registration is affected by delays in or lack of birth or death notification.

Governments are starting to use mobile devices such as mobile phones to reduce problems with birth or death notification. In some settings, members of the public, healthcare workers and others use mobile phones to notify a birth or death to the health system or to a central registration system.

The main aim of our review was to assess what happens when people use mobile devices to notify births and deaths, compared to other systems or no systems at all. For instance, do they register more birth and deaths, and do they do this at the right time? And does this lead more babies and children to use or receive health services, such as immunisation? We also wanted to find out how people are using these mobile systems in practice and what influences their use.

**What are the main results of the review?**

We found 21 relevant studies. All of the studies were from low‐ or middle‐income countries. Most of these studies focussed on the notification of births and deaths in rural, remote, or marginalised populations who are often under‐represented in birth or death registration processes or have poor access to health services. Only one of the studies assessed the effect of using mobile devices for notification systems. This study focussed on birth notification. We did not find any studies that assessed the effect of using mobile devices for death notification. We are uncertain of the effect of this approach on the number of births and deaths that are properly notified because the certainty of this evidence was assessed as very low.

The other studies had information about how people use the mobile device‐based birth and death notification systems in practice. These studies pointed to several factors that could influence the implementation of birth‐death notification via mobile devices. These factors were tied to the health system and the notification system, the person responsible for notifying, the community, and the families involved. They include:

‐ Geographic barriers that could prevent people’s access to birth‐death notification and post‐notification services

‐ Access to health workers and other notifiers with enough training, supervision, support, and incentives

‐ Monitoring systems that ensure the quality and timeliness of the birth and death data

‐ Legal frameworks that allow births and deaths to be notified by mobile device and by different types of notifiers

‐ Community awareness of the need to register births and deaths

‐ Socio‐cultural norms around birth and death

‐ Government commitment

‐ Cost to the system, to health workers and to families

‐ Access to electricity and network connectivity, and compatibility with existing systems

‐ Systems that protect data confidentiality

We have low to moderate confidence in these findings. This was mainly because of the ways in which the studies were designed and small amounts of data.

**How up‐to‐date is this review?**

We searched for studies that had been published up to August 2, 2019.

## Summary of findings

**Summary of findings 1:** Primary objective: Summary of findings

**Birth notification via mobile device compared to standard practice**				
**Patient or population**: Health Care Workers (HCWs), Village Health Volunteers (VHVs), newborn children **Setting**: Lao People’s Democratic Republic **Intervention**: Provision of mobile phone and credit to HCWs and VHVs to facilitate birth notification **Comparison**: Standard practice, i.e. no provision of mobile phone or credit to HCWs and VHVs to facilitate birth notification				
**Outcomes**	**Birth notification via mobile phone versus standard practice**	**No of Participants** **(studies)**	**Certainty of the evidence** **(GRADE)**	**What happens?**
**Coverage of birth notification**				
Proportion of VHVs who reported notifying a HCW about deliveries or births using mobile phones (post‐intervention comparison)	12% more VHVs in the intervention group reported notifying a HCW using mobile phones compared to the comparison group	101 (1 CBA)^1^	⊕⊝⊝⊝ VERY LOW^2,3,4^	We are uncertain of the effect of the intervention on coverage of birth notification because the certainty of the evidence is very low.
Proportion of HCWs who reported receiving a notification from VHV about deliveries or birth using mobile phones (post‐intervention comparison)	38% more HCWs in the intervention group reported receiving a notification using mobile phones compared to the comparison group	30 (1 CBA)^1^	⊕⊝⊝⊝ VERY LOW^2,3,4^	
**Timeliness of birth notification**				
Proportion of VHVs who reported notifying HCWs either during labour or within 1 day of birth using mobile phones	18% moreVHVs in the intervention group reported notifying HCWs of imminent deliveries within 1 day of birth via mobile phones compared to the comparison group	101 (1 CBA)^1^	⊕⊝⊝⊝ VERY LOW^2,3,4^	We are uncertain of the effect of the intervention on the timeliness of birth notification because the certainty of the evidence is very low.
Proportion of HCWs who reported receiving a notification from VHV about imminent deliveries or within 1 day of birth using mobile phones	15% moreHCWs in the intervention group reported being notified by VHVs of imminent deliveries within 1 day of birth via mobile phones compared to the comparison group	30 (1 CBA)^1^	⊕⊝⊝⊝ VERY LOW^2,3,4^	
**Proportion and timeliness of legal birth registrations**				
No studies were identified that reported on this outcome.				
**Coverage of newborn or child health services**				
Proportion of births where HCW made postnatal care visit to home	There were 10% more postnatal care home visits by HCW in the intervention group compared to the comparison group.	1339 (1 CBA)^1^	⊕⊝⊝⊝ VERY LOW^2,3,4^	We are uncertain of the effect of the intervention on coverage of newborn or child health services because the certainty of the evidence is very low
Proportion of births for which Hepatitis B birth dose vaccination was provided within 30 days	There were 23% more children who received the Hepatitis B birth dose vaccination within 30 days of birth in the intervention group compared to the comparison group	1525 (1 CBA)^1^	⊕⊝⊝⊝ VERY LOW^2,3,4^	
**Timeliness of newborn or child health services**				
Proportion of births where Hepatitis B birth dose vaccination was administered within 0‐1 day	There was a 0% change in the number of newborns receiving Hepatitis B birth dose vaccination within the first day after birth in the intervention group compared to comparison group.	1525 (1 CBA)^1^	⊕⊝⊝⊝ VERY LOW^2,3,4^	We are uncertain of the effect of the intervention on timeliness of newborn or child health services because the certainty of the evidence is very low
Proportion of births where Hepatitis B birth dose vaccination was administered within 2‐7 days	5% fewer children received Hepatitis B birth dose vaccination between days 2 and 7 in the intervention group compared to the comparison group.	1525 (1 CBA)^1^	⊕⊝⊝⊝ VERY LOW^2,3,4^	
Proportion of births where the HCW made a postnatal care home visit within 24 hours of notification	18% fewer children received a postnatal care visit at least 50% of the time by the HCW in the intervention group within 24 hours of birth compared to the comparison group.	30 (1 CBA)^1^	⊕⊝⊝⊝ VERY LOW^2,3,4^	
*The basis for the **assumed risk** (e.g. the median control group risk across studies) is provided in footnotes. The **corresponding risk** (and its 95% confidence interval) is based on the assumed risk in the comparison group and the **relative effect** of the intervention (and its 95% CI). **CI:** Confidence interval; **RR:** Risk Ratio; **CBA**: Controlled Before‐After study; **VHV**: Village Health Volunteer; **HCW**: Health Care Worker				
GRADE Working Group grades of evidence **High** = This research provides a very good indication of the likely effect. The likelihood that the effect will be substantially different^†^ is low. **Moderate** = This research provides a good indication of the likely effect. The likelihood that the effect will be substantially different^†^ is moderate. **Low** = This research provides some indication of the likely effect. However, the likelihood that it will be substantially different^†^ is high. **Very low** = This research does not provide a reliable indication of the likely effect. The likelihood that the effect will be substantially different^†^ is very high. ^†^ Substantially different = a large enough difference that it might affect a decision				

^1^Xeuatvongsa 2016 **Explanations for certainty rating:** ^2^ Initial rating of low certainty assigned due to non‐randomised study design, resulting in high or unclear risk of bias ^3^ The initial rating was downgraded one level to very low certainty for outcomes related to the coverage and timeliness of birth notification due to small sample sizes and small numbers of events. ^4^ The initial rating was downgraded one level to very low certainty for outcomes related to the coverage and timeliness of post‐notification health services due to concerns related to indirectness. It is unclear how many of the post‐notification service events were directly in response to the notification. **Abbreviations:** VHV, Village Heath Volunteer; HCW, Health Care Worker.

**Summary of findings 2:** Secondary objectives: Summary of qualitative findings

**Summary of review finding**		**Studies contributing to the review finding**	**Overall GRADE‐CERQual assessment of confidence in the evidence**	**Explanation for assessment**	
**A. Health system constraints in the implementation of birth and death notification via mobile devices**					
**A.1**	Geographic barriers hamper timeliness of birth and death notification conducted via mobile devices, as well as post‐notification services or processes (e.g. certification of birth or death).	Xeuatvongsa 2016; ANISA 2016; Pascoe 2012; MOVE‐IT 2013; Ngabo 2012; MBRT 2016, mVRS 2017	**Moderate confidence**	Serious concerns related to methodological limitations. Few or no concerns related to coherence, relevance and adequacy	
**A.2**	Birth and death data collected using mobile devices can help health and civil registration systems identify problems and introduce appropriate quality improvements.	Moshabela 2015; MBRT 2016; RapidSMS 2012; MVH 2017; NIMDS 2019	**Low confidence**	Serious concerns related to methodological limitations and adequacy. Few or no concerns with coherence and relevance	
**A.3**	Health workers who lack familiarity with, or prior experience in, using mobile technologies may need rigorous training as well as post‐training support.	Andreatta 2011; Gisore 2012; Ngabo 2012; mSIMU 2017; Yugi 2016 mTika 2016; Xeuatvongsa 2016; MBRT 2016; Van Dam 2015; MBRL 2011; MOVE‐IT 2013; NIMDS 2019	**Moderate confidence**	Moderate concerns related to methodological limitations. Few or no concerns related to coherence, relevance, and adequacy	
**A.4**	Local capacity to train future cadres of notifiers may be strengthened though 'train the trainer' approaches.	Ngabo 2012; MBRL 2011	**Low confidence**	Serious concerns related to methodological limitations and adequacy. Few or no concerns with coherence and relevance	
**A.5**	Mechanisms for continuous monitoring and supportive supervision are important for ensuring the quality and timeliness of birth and death data collected via mobile devices.	Andreatta 2011; mTika 2016; Ngabo 2012; MOVE‐IT 2013; Yugi 2016; Pascoe 2012	**Moderate confidence**	Moderate concerns related to methodological limitations and adequacy. Few or no concerns with coherence and relevance	
**A.6**	Inadequate attention is paid to legal frameworks governing civil registration. These may need to be modified to allow notification via mobile device and the inclusion of new cadres of notifiers (low confidence finding).	eCRVS‐Mozambique 2017; mVRS 2017; MBRP 2015	**Low confidence**	Serious concerns related to methodological limitations and adequacy. Few or no concerns with coherence and relevance	
**A.7**	The availability of adequate human resources to conduct birth and death notification via mobile devices may be facilitated by hiring new cadres of notifiers or recruiting existing cadres of health workers to undertake notification.	Andreatta 2011; MBRL 2011; Gisore 2012; Pascoe 2012; MOVE‐IT 2013; Xeuatvongsa 2016; Yugi 2016; ANISA 2016; mVRS 2017; eCRVS‐Mozambique 2017; MBRT 2016	**Moderate confidence**	Serious concerns related to methodological limitations. Few or no concerns with coherence, relevance, and adequacy	
**A.8**	Implementing birth and death notification via mobile devices may be influenced by underlying health and civil registration system infrastructure, resources, and processes.	Ngabo 2012; MBRL 2011; MOVE‐IT 2013; Moshabela 2015; ANISA 2016; mVRS 2017; Gisore 2012;MVH 2017	**Low confidence**	Serious concerns related to methodological limitations. Minor concerns related to adequacy. Few or no concerns with coherence, and relevance	
**B. Factors related to individuals providing birth and death notification via mobile devices**					
**B.1**	Costs incurred by health workers sending notification using personal mobile phones may need to be reimbursed to facilitate sustained use of these technologies for notification.	Ngabo 2012; Pascoe 2012; mSIMU 2017; Yugi 2016; Xeuatvongsa 2016; Gisore 2012	**Moderate confidence**	Moderate concerns related to methodological limitations and adequacy. Few or no concerns related to coherence or relevance	
**B.2**	The use of mobile phones for notification is acceptable to health workers, and helps them to undertake their job responsibilities.	Ngabo 2012; Pascoe 2012; mSIMU 2017, Van Dam 2015; Yugi 2016; NIMDS 2019	**Moderate confidence**	Moderate concerns related to methodological limitations and adequacy. Few or no concerns related to coherence and relevance	
**B.3**	Health workers’ adoption of mobile birth and death notification strategies may be affected by competing priorities and the availability of adequate incentives.	MOVE‐IT 2013; mSIMU 2017; mTika 2016; MVH 2017	**Moderate confidence**	Minor concerns related to methodological limitations. Serious concerns related to adequacy. Few or no concerns related to coherence and relevance	
**C. Factors related to families for whom birth and death is notified via mobile devices**					
**C.1**	For some families, costs may be a barrier to completing birth and death registration post‐notification.	MOVE‐IT 2013, MBRP 2015, MBRT 2016	**Low confidence**	Serious concerns related to methodological limitations and adequacy. Few or no concerns related to coherence, relevance, and adequacy	
**C.2**	There may be a need for targeted demand generation activities in communities with low awareness of the need of birth and death registration, alongside the use of mobile phones for birth and death notification.	MOVE‐IT 2013; MBRG 2014; mVRS 2017; MBRT 2016;	**Low confidence**	Serious concerns related to methodological limitations. Moderate concerns related to adequacy. Few or no concerns related to coherence and relevance	
**C.3.**	Sociocultural norms may influence the timely identification of births and deaths, and should be taken into consideration when developing mobile phone interventions for notification.	MOVE‐IT 2013; MBRG 2014; MBRP 2015; ANISA 2016	**Low confidence**	Serious concerns related to methodological limitations and adequacy. Few or no concerns related to coherence and relevance	
**C.4**	Birth and death notification may increase access to these services for some families. However, they may also increase inequities in access related to low availability of supportive infrastructure (network coverage, roads, human resources), human factors (age, gender, literacy, poverty), and selective funding priorities of donors.	Gisore 2012; MBRP 2015; MBRT 2016; Andreatta 2011; Ngabo 2012; MOVE‐IT 2013; mSIMU 2017; mTika 2016; Yugi 2016; Xeuatvongsa 2016; mVRS 2017	**Moderate confidence**	Serious concerns related to methodological limitations. Few or no concerns related to coherence, relevance, and adequacy	
**D. Factors related to government involvement in birth and death notification via mobile devices**					
**D.1**	Strong government commitment is a key factor in the successful implementation of birth and death notification via mobile devices.	MBRL 2011; Ngabo 2012; Yugi 2016; mVRS 2017; eCRVS‐Mozambique 2017; MBRT 2016; MBRP 2015	**Low confidence**	Serious concerns related to methodological limitations. Moderate concerns related to adequacy. Few or no concerns related to coherence or relevance	
**E. Factors related to technologies used for birth and death notification via mobile devices**					
**E.1**	Cost is an important consideration in the purchase, set‐up, and scaling up of mobile technologies needed for birth and death notification.	Ngabo 2012; Xeuatvongsa 2016; Gisore 2012; mTika 2016; Pascoe 2012; Van Dam 2015; Yugi 2016; mVRS 2017	**Low confidence**	Serious concerns related to methodological concerns. Moderate concerns related to adequacy. Few or no concerns related to coherence and relevance	
**E.2**	Challenges when notifying births and deaths via mobile devices include poor access to electricity and incompatibility with existing systems.	Ngabo 2012; MBRL 2011; Pascoe 2012; Gisore 2012; MBRP 2015; MBRT 2016	**Low confidence**	Serious concerns related to methodological concerns. Moderate concerns related to adequacy. Few or no concerns related to coherence and relevance	
**E.3**	The availability of network connectivity is a key factor in the successful implementation and scale‐up of birth and death notification via mobile devices.	Ngabo 2012; Pascoe 2012; Yugi 2016; ANISA 2016; mSIMU 2017; mVRS 2017; MBRT 2016,	**Moderate confidence**	Serious concerns related to methodological limitations. Few or no concerns with coherence, relevance, and adequacy	
**E.4**	Data security and encryption measures are needed to preserve confidentiality of birth and death information notified via mobile devices.	Van Dam 2015; MBRT 2016; Ngabo 2012; MVH 2017	**Low confidence**	Serious concerns with methodological limitations and adequacy. Few or no concerns with coherence and relevance	

## Background

Globally, the birth of nearly 230 million children under the age of five, and two‐thirds of all deaths have not been officially registered (UNICEF 2016; WHO 2017a; World Bank 2014). Birth registration is a child’s right, and serves as the foundation for establishing legal identity, equitable access to basic services such as healthcare and education, and protection from exploitation (UNHCR & UNICEF 2017; UNICEF 2013). Similarly, death registration, including identification of cause of death, enables public health systems to develop and implement programmes to improve the health of populations, as well as rapidly deal with outbreaks (WHO 2013a; WHO 2017a; World Bank 2014). In the context of the post‐2015 development agenda, timely, accurate, and complete statistics on births and deaths, gained through the act of registration, are fundamental for tracking progress towards sustainable development goals and achievement of universal health coverage (WHO 2017b).

### Description of the condition

Well‐functioning Civil Registration and Vital Statistics systems provide the most reliable and up‐to‐date data on births, deaths, and population size (UN‐DECA 2014). Civil registration is defined as the "'universal, continuous, permanent, and compulsory recording of vital events (live births, deaths, fetal deaths, marriages, and divorces) provided through decree or regulation in accordance with the legal requirements of each country" (UN‐DECA 2002; UNHCR & UNICEF 2017). Vital statistics are the compilation, processing, and dissemination of civil registration data in statistical form (Setel 2007; UN‐DECA 2014; UN‐DECA 2017). Statistics on births and deaths are used to generate population health indicators (e.g. fertility rate, birth rate, and life expectancy), data on mortality (e.g. maternal and infant mortality rates), and disease burden (e.g. using details of cause of death (UN‐DECA 2014)). Hence, birth and death statistics are a valuable source of data for policymakers, to guide the development of global, national, and regional health policy, programme planning, and appropriate resource‐allocation (Setel 2007; UN‐DECA 2014).

Over 100 developing countries lack functional or adequate civil registration systems for capturing vital events (World Bank 2014). The majority of individuals missed by civil registration systems reside in South Asia and sub‐Saharan Africa (AbouZahr 2015; Setel 2007; UNICEF 2016). Birth and deaths of individuals living in rural areas, or lower socioeconomic status households, are more likely to be unregistered, compared to their urban and wealthier counterparts (UNICEF 2013). There is also a link between birth registration and health outcomes (Phillips 2015). For example, children who are unregistered are more likely to miss out on essential health services, such as immunisations (Apland 2014; Fagernas 2013). Lack of accurate and timely death statistics, including cause of death, leads to weak disease surveillance, and threatens the ability of public health systems to prevent or rapidly deal with outbreaks (UN‐DECA 2017). From the health system perspective, the paucity of accurate statistics on births and deaths poses a key challenge in the estimation of programme needs (e.g. number of children eligible for health services), appropriate resource allocation, and monitoring (e.g. for calculation of indicators of health system coverage or performance (AbouZahr 2015; AbouZahr 2015a; Mahapatra 2007)).

Several challenges to civil registration have been identified in the literature, including geographic barriers (UNICEF 2013), low demand or lack of incentives for registration (Apland 2014; UNICEF 2013; WHO 2013b; World Bank 2014), use of paper‐based systems for reporting and recording births (Oomman 2013; World Bank 2014), and lack of, or incorrect, cause of death coding and documentation (Mikkelsen 2015; Rampatige 2013). Poor integration of Civil Registration and Vital Statistics systems with other government or citizen databases leads to missed opportunities, for instance, where data on births and deaths captured by the health system are not linked to civil registration systems (World Bank 2014). Even when integration between the health and civil registration system may exist, home births or deaths may not be reported where formal community‐level notification processes are deficient (World Bank 2014).

A global scale‐up plan for strengthening civil registration systems has been developed by the World Health Organization and the World Bank, with the aim to "achieve universal civil registration of births, deaths, and other vital events, including reporting cause of death, and access to legal proof of registration for all individuals by 2030” (World Bank 2014). A cornerstone of this plan is the prioritisation and strengthening of the linkages between health and Civil Registration and Vital Statistics systems (Muzzi 2010; WHO 2013a; World Bank 2014). This includes a push to modernise data systems associated with civil registration through the use of digital information systems, and to improve coverage of registration services among underserved populations such as those residing in rural areas (Oomman 2013; World Bank 2014). In these respects, the global proliferation of mobile phones and cellular network connectivity is increasingly being leveraged, especially in resource‐limited settings, to drive development and use of digital civil registration applications (ITU 2016; Labrique 2012; Labrique 2013; Oomman 2013). Official notifiers include health workers or other cadres of workers permitted under law to carry out notifications. With growing access to mobile phones, community‐based individuals, such as vaccination programme workers, community health workers, and village elders can serve as 'notifiers', helping to increase the reach of civil registration systems to underserved rural and remote regions (World Bank 2014). Such an approach may help to reduce delays in identification and reporting of births and deaths to health systems, local civil registration authorities, or both (World Bank 2014).

### Description of the intervention

Civil registration involves four major activities: recording, notification, registration, and certification (see Figure 1 (Setel 2007; WHO 2013a)).

**1:**
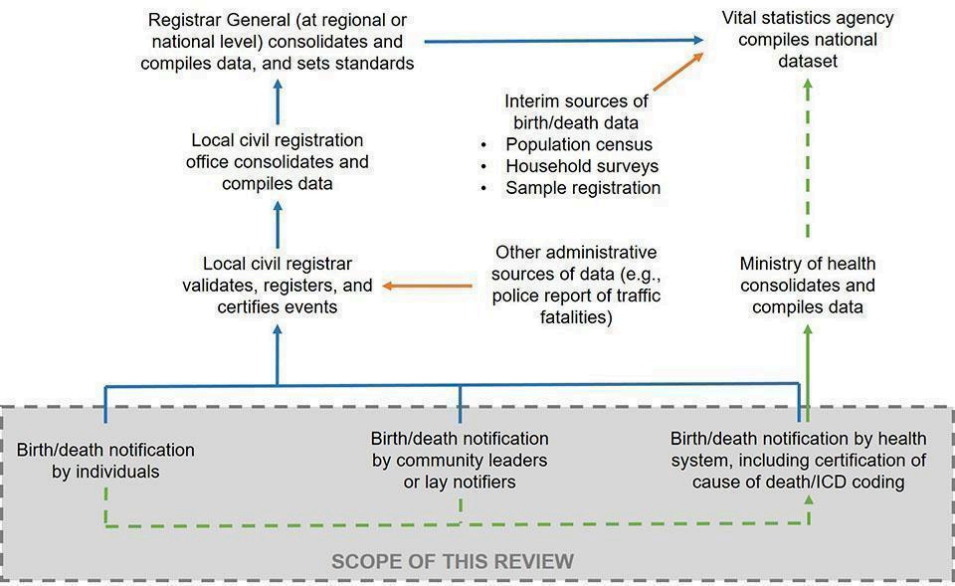
**Linkage between CRVS and health system** *Adapted from*Setel 2007*and World Bank and World Health Organization 2014 (*World Bank 2014)*.*

- Recording entails capturing details related to a vital event at the point of the event. For example, details of a birth may be recorded on a paper form at the health facility or at home.
- This is followed by notification, wherein details of the recorded event are communicated to the local civil registration office by lawful notifiers. In official terms, a notification is defined as the capture and onward transmission of minimum essential information on the fact of birth or death by a designated informant, agent or official of the CRVS system using a CRVS authorised notification form (paper or electronic) with that transmission of information being sufficient to support eventual registration and certification of the vital event.
- Upon receiving a notification, the civil registrar registers the event, by verifying event details, and recording them in a civil register.
- Subsequently, a legally valid certificate of registration is issued. The certificate serves as proof that the birth or death has been registered in a civil register.
- Registered events are aggregated by the national authorities to produce vital statistics on key health and development indicators.

Since notification is the key step that triggers registration, many strategies to improve the coverage and timeliness of birth or death registration are focussed on reducing delays in notification, especially by using mobile devices to notify local officials. The scope of this review is limited to the notification of births and deaths conducted via mobile devices. 

- By **birth notification**, we mean the transmission of information via a mobile device to a centralised system or focal individual(s) to report a birth event.
  - In addition to the formal notification process, which leads to birth registration as it occurs within the context of Civil Registration and Vital Statistics systems, we included informal notification of births in this definition. By this, we mean that individuals, other than those defined under the law as official notifiers, may be involved in notifying with mobile devices. It may also mean that the notification is directed to focal individuals other than the civil registrar, or communicated directly to a digital system, and transmitted for purposes other than civil registration.
- By **death notification**, we mean the transmission of information via a mobile device to a centralised system or focal individual(s) to report a death event. Death notification may include information on the cause of death.
  - As in the case of birth notification, we also included informal notifications of death in this definition. By this, we mean that individuals, other than those defined under the law as official notifiers, may be involved in providing a notification. It may also mean that the notification is directed to focal individuals other than the civil registrar, or communicated directly to a digital system, and may be transmitted for purposes other than civil registration.
- By **mobile devices**, we mean mobile phones of any kind (but not analogue land line telephones), as well as tablets, personal digital assistants, and smartphones. Laptops are not included in this list.

### How the intervention might work

For **birth notifications**, information related to the birth may be transmitted via mobile phones as phone calls, inputs to an interactive voice response, or an unstructured supplementary service data (USSD) system, as short messaging service (SMS), from mobile device‐based applications (apps), or to publicly known short codes or access numbers. The content of the birth notification may vary by country or implementation, but may include the name of the child born, name and address of the parents, place and date of birth, and details of birth outcomes.

- An example of a **formal birth notification** sent via a mobile device, is when a community‐based notifier uses his or her mobile phone to relay notification about a home‐based birth to a digital civil registration system via USSD (NIRA 2017). The notification may be received and reviewed for accuracy and completeness by the local civil registration office before a birth certificate is issued. Direct notification to the civil registrar by lawful notifiers is considered an active notification. Passive notification occurs in cases where a notification form is provided by health authorities to families and when family members bear the onus of reporting the birth or death event to the civil registrar.
- An example of an **informal birth notification** sent via a mobile device, is when a village elder sends information about a birth, via SMS, to a central digital server, for the purpose of enroling the child in a longitudinal vaccination tracking system. The enrolment of the child in the tracking system may be used to initiate vaccination services for the child, and to track their subsequent vaccinations.

For **death notifications**, information related to the death may be transmitted via mobile phones as phone calls, inputs to an interactive voice response or USSD system, as SMS, from apps, or to publicly known short codes or access numbers. The content of the death notification may vary by country or implementation, but may include name of the deceased, name and address of relatives (for example, spouse), place and date of death, and details of the cause of death.

- An example of a **formal death notification**, sent via mobile device, is when a health worker uses a mobile phone app to transmit information about a death, including cause of death, to a digital civil registration system. The notification may be received and reviewed for accuracy and completeness by the local civil registration office before a death certificate is issued.
- An example of an **informal death notification** sent via a mobile device, is when a community health worker sends a message about a death, via SMS, to a central digital server, for the purpose of disease surveillance.

### Why it is important to do this review

Ministries of health, donors, and decision‐makers face expanding opportunities to harness the ubiquity and penetration of mobile technology to address longstanding challenges related to acquiring accurate and timely statistics on births and deaths. There is high demand from these stakeholders for evidence‐based guidance on the value of digital tools to strengthen linkages between civil registration and health systems, as a mechanism to improve the timeliness and accuracy of birth and death statistics. In response to this global need, the World Health Organization has developed guidelines to inform investments on digital health approaches that use mobile phones for birth and death notifications (WHO Guidelines 2019).

There is growing evidence on the use of mobile devices for birth and death notification. A previous systematic review on digital interventions for Civil Registration and Vital Statistics was published in 2013 (WHO 2013a). It examined literature from 23 countries, but found limited peer‐reviewed evidence for the use of mobile devices to notify birth and death events. This review, focussed entirely on low‐ and middle‐income countries, did not report quantitative outcomes, or examine factors that influenced the use of mobile phones to notify officials of birth and death events. Since this review was published, several new studies describing birth or death notification via mobile devices have emerged. Hence, it is important to conduct a systematic review to assess these new studies. Preliminary findings from this systematic review were used to directly inform WHO guidelines on the effectiveness of digital strategies to improve data on births and deaths (WHO Guidelines 2019).

## Objectives

### Primary objectives

- To assess the effects of birth notification via a mobile device, compared to standard practice.
- To assess the effects of death notification via a mobile device, compared to standard practice.

### Secondary objectives

- To describe the range of strategies used to implement birth and death notification via mobile devices.
- To identify factors influencing the implementation of birth and death notification via mobile devices.

## Methods

### Criteria for considering studies for this review

#### Types of studies

To address the **primary objectives**, we included the following study designs:

- Individual and cluster‐randomised trials;
- Cross‐over and stepped‐wedge study designs;
- Controlled before‐after studies, provided they had at least two intervention sites and two control sites; and
- Interrupted time series studies, if there was a clearly defined time point when the intervention occurred and at least three data points before and three after the intervention.

To address the **secondary objectives**, we included any study design, either quantitative, qualitative, or descriptive, that aimed to:

- Describe current strategies for birth and death notification via mobile devices; or
- Explore factors that influence the implementation these strategies, including studies of acceptability or feasibility.

To address both the primary and secondary objectives, we included published studies, conference abstracts, and unpublished data. We included studies regardless of their publication status and language of publication.

#### Types of participants

The following participants were included in this review:

- All cadres of healthcare providers, including professionals, paraprofessionals, and lay health workers (LHWs);
- Administrative, managerial, and supervisory staff at health facilities;
- Administrative, managerial, and supervisory staff, including registrars, associated with civil registration units;
- Focal individuals at the village‐ or community‐level (e.g. village leaders);
- Parents or other caregivers (e.g. grandparents) of children whose birth is being notified; and
- Relatives or caregivers of deceased individuals.

#### Types of interventions

To address the **primary objectives**, we included studies that compared birth and death notification via mobile devices with standard practice. We defined standard practice as non‐digital and non‐mobile, paper‐based processes and workflows for notifying birth and death events.

The comparisons for this review were:

- birth notification via mobile devices compared with standard practice; and
- death notification via mobile devices, compared with standard practice.

We included:

- studies in which birth or death notification was sent by parents, caregivers, other family members, administrative, managerial or supervisory staff, focal individuals in the community, or health workers, via mobile devices, to alert a central system, organisation, or civil registration agency that a birth or death has taken place;
- studies in which notified births were enrolled into a digital health record for tracking provision of newborn and child health services;
- studies in which birth notification was part of a pregnancy digital health record, and where outcomes were reported for the postnatal period onward;
- studies in which notified deaths, including cause of death, were reported to a disease surveillance system; and
- studies in which birth and death notifications were delivered as part of a wider package, if we judged the birth or death notification to be the major component of the intervention.

To address the **secondary objectives**, in addition to the above inclusion criteria, we included:

- studies in which birth and death notifications were delivered as part of a wider package:

  - even if birth and death notifications were judged not to be the major component of the intervention; and
  - as long as we could extract data on the birth and death notification components that were relevant to the secondary objectives.

When addressing both the primary and secondary objectives, we excluded:

- studies in which birth and death notification was conducted on stationary computers or laptops alone;
- studies that compared different specifications of technology systems (e.g. software, communication channels) for birth or death notification;
- studies in which birth notification was part of a pregnancy digital health record, and where outcomes were only reported for the pregnancy period. Such studies were excluded from this review because we would not be able to link the effect of the mobile birth notification to outcomes that occurred during pregnancy. While such studies were excluded from this review, outcomes related to the pregnancy period from such studies were extracted and included in a separate review.
- studies that only described interventions to improve attribution of cause of death (e.g. digital verbal autopsy tools), without a notification component; and
- feasibility or pilot studies (for the primary objectives only. These study designs were included for the secondary objectives).

#### Types of outcome measures

##### Primary objective: Types of outcome measures

To address the **primary objectives**, we included studies that reported outcomes related to birth and death notification via mobile devices. When birth and death notifications were described in the same study, we extracted and reported outcome data for birth and death notifications separately. Specific outcomes of interest are listed below.

###### For birth notification via mobile device

- coverage (e.g. proportion) of births notified via mobile devices;
- timeliness of birth notification via mobile device (e.g. time between birth and birth notification via mobile device);
- proportion of legal birth registrations in response to birth notifications via mobile device, where legal birth registration is defined as the recording, within the civil registry, of the occurrence and characteristics of births in accordance with the legal requirements of a country. Legal birth registration is conducted by a civil registrar.
- timeliness of legal birth registrations in response to birth notification via mobile device (e.g. time between birth notification and legal birth registration);
- coverage of (e.g. proportion of children receiving) newborn or child health services (e.g. immunisations) in response to birth notification via mobile device;
- timeliness of receipt of newborn or child health services (e.g. immunisations) in response to birth notification via mobile device (i.e. time between birth and receipt of services).

###### For death notifications via mobile device

- coverage (e.g. proportion) of deaths notified via mobile devices;
- timeliness of death notification via mobile device (i.e. time between death and death notification via mobile device);
- proportion of legal death registrations in response to death notifications via mobile device, where legal death registration is defined as the recording, within the civil registry, of the occurrence and characteristics of death in accordance with the legal requirements of a country. Legal death registration is conducted by a civil registrar.
- timeliness of legal death registrations in response to death notification via mobile device (i.e. time between death notification and legal death registration);
- proportion of deaths where causes of death were ascertained, reported, or both, to a disease surveillance system in response to death notifications via mobile device;
- timeliness of causes of death ascertainment, reporting to a disease surveillance system, or both, in response to death notifications via mobile device (i.e. time between death and cause of death ascertainment).

###### For both birth and death notifications via mobile device

- quantitative measures of notifiers’ acceptability or satisfaction (or both) with birth and death notifications via mobile device;
- resource use (e.g. human resources and time, including additional time spent by notifiers when managing and transitioning from paper to digital reporting systems, training, supplies, and equipment);
- unintended consequences (e.g. transmission of inaccurate data, for instance, by incorrect data entry, privacy and disclosure issues, failure or delay in message delivery, interrupted workflow due to infrastructure constraints for recharging batteries and network coverage, and impact on equity).

##### Secondary objectives: Topics of interest

To address the **secondary objectives**, we extracted data about strategies for the notification of births and deaths via mobile devices, and data about factors that influenced the implementation of these strategies.

### Search methods for identification of studies

An independent information specialist (JE) developed the search strategies in consultation with the review authors. We only included studies published after 2000. This decision was based on the increased availability and penetration of mobile devices in low‐ and middle‐income countries starting in 2000 (ITU 2016). Search strategies were comprised of titles, abstracts, and keywords, including controlled vocabulary terms. We did not apply any limits on language.

We used a study design search filter used by Cochrane Effective Practice and Organisation of Care (EPOC) to retrieve both randomised and non‐randomised studies. See Appendix 1 for all search strategies used.

#### Electronic searches

To address the **primary and secondary objectives**, we searched the following databases:

- Cochrane Central Register of Controlled Trials (CENTRAL) in the Cochrane Library (Issues 8, 2019, searched on August 2, 2019)
- MEDLINE and Epub Ahead of Print, In‐Process & Other Non‐Indexed Citations and Daily 1946 to August 01, 2019, Ovid (searched on August 2, 2019)
- Embase 1974 to 2019 Week 30, Ovid (searched on August 2, 2019)
- Global Index Medicus/Global Health Library, WHO (searched on August 2, 2019)
- POPLINE K4Heath (searched on August 2, 2019)

#### Searching other resources

To address both the **primary and the secondary objectives**, we also searched the following sources:

##### Trial registries

- World Health Organization International Clinical Trials Registry Platform (WHO ICTRP; www.who.int/ictrp, searched on August 2, 2019);
- US National Institutes of Health Ongoing Trials Register ClinicalTrials.gov (www.clinicaltrials.gov, searched on August 2, 2019).

##### Systematic review registry

We searched Epistemonikos (www.epistemonikos.org) on September 27, 2019 for related systematic reviews and potentially eligible primary studies.

##### Grey literature

We conducted a grey literature search to identify studies not indexed in the databases listed above, and to capture the broader range of study designs to be included for the secondary objectives. Because this review is focussed on birth and death notifications using mobile devices, we reviewed mhealthevidence.org on August, 15, 2017 for contributed content that is not referenced in MEDLINE Ovid. In addition, the WHO issued a call for papers through popular digital health communities of practice, such as the Global Digital Health Network and Implementing Best Practices, to identify additional primary studies and grey literature. Results from the grey literature were only incorporated in the first round of the search since the mhealthevidence.org database was no longer being curated at the time of subsequent searches.

##### Other resources

- We reviewed reference lists of all included studies and relevant systematic reviews for potentially eligible studies.
- We contacted authors of included studies and reviews to clarify reported published information, and to seek unpublished results and data.
- We conducted citation searches of included studies in Web of Science, Clarivate Analytics; and in Google Scholar (searched on May 15, 2020).

### Data collection and analysis

#### Selection of studies

We downloaded all titles and abstracts retrieved by electronic searching to a reference management database, and removed duplicates. Two review authors independently screened titles and abstracts for inclusion. We retrieved the full‐text study reports and publications, and two review authors independently screened the full texts, identified studies for inclusion, and identified and recorded reasons for excluding ineligible studies. We resolved any disagreement through discussion or, if required, we consulted a third review author. For one study in French, we consulted with a review author with appropriate fluency.

We listed studies that initially appeared to meet the inclusion criteria, but that we excluded after reviewing the full‐text report, in the Characteristics of excluded studies table. We collated multiple reports of the same study so that each study, rather than each report, was the unit of interest in the review. We also recorded any information that we could obtain about relevant ongoing studies. We recorded the selection process in sufficient detail to complete a PRISMA flow diagram (Liberati 2009).

#### Data extraction and management

We used the EPOC standard data collection form and adapted it for study characteristics and outcome data (EPOC 2017a); we piloted the form on at least one study in the review.

To address the **primary objectives**, two review authors independently extracted the study characteristics from the included studies, including:

1. General information: title, reference details, author contact details, publication type, funding source, conflicts of interest of study authors;
2. Methods: study design, number of study sites and location, study setting, withdrawals, date of study, follow‐up;
3. Participants: number, mean age, age range, gender, severity of condition, inclusion criteria, exclusion criteria, other relevant characteristics;
4. Interventions: intervention components, comparison, intervention purpose, mode, timing, frequency, and duration of intervention delivery, content of the intervention, type of mobile device used (smartphone, tablet, feature phone, basic phone), interoperability, compliance with national guidelines, data security, fidelity assessment;
5. Outcomes: main and other outcomes specified and collected, time points reported;
6. Notes: funding for trial, notable conflicts of interest of trial authors, ethical approval, interoperability, data security, compliance with national guidelines, limitations for delivery at scale.

Two review authors independently extracted outcome data from included studies. We noted in the Characteristics of included studies table if outcome data were reported in an unusable way. We resolved disagreements by consensus or by involving a third review author.

To address the first of the **secondary objectives** on describing the range of strategies to used to implement birth and death notification via mobile devices, one review author extracted descriptive data where applicable and available, including the details of the intervention/s used, groups or stakeholders involved in implementing the intervention, pathway of action (how they thought it would work), context of implementation, type of evaluation (study design), and outcome measures assessed. A second review author checked the extracted data.

To address the second of the **secondary objectives** on assessing the factors affecting the implementation of birth and death notifications via mobile device, one review author used the SURE (Supporting the Use of Research Evidence) framework (Appendix 2), which provides a comprehensive list of possible factors that may influence the implementation of health system interventions (Glenton 2017; SURE 2011). A second review author checked the extracted data. We extracted data on:

1. health system constraints (e.g. accessibility of care, financial resources, human resources, educational and training system, including recruitment and selection, clinical supervision, support structures and guidelines, internal communication, external communication, allocation of authority, accountability, community participation, management or leadership (or both), information systems, facilities, client processes, distribution systems, incentives, bureaucracy, relationship with norms and standards)
2. individual characteristics (e.g. knowledge and skills, attitudes regarding programme acceptability, appropriateness and credibility, motivation to change or adopt new behaviour)
3. social and political constraints (e.g. ideology, governance, short‐term thinking, contracts, legislation or regulation, donor policies, influential people, corruption, political stability and commitment)

In addition, we included any emergent codes which were not captured within the SURE framework but that described implementation challenges.

#### Assessment of risk of bias in included studies

##### Assessment of risk of bias in included studies for the primary objective

For studies addressing the **primary objectives**, two review authors independently assessed risk of bias, using the criteria outlined in the *Cochrane Handbook for Systematic Reviews of Interventions* (Higgins 2011), and the guidance from the EPOC group (EPOC 2017b). Any disagreements were resolved by discussion, or by involving a third review author. We assessed the risk of bias according to the following domains:

1. random sequence generation;
2. allocation concealment;
3. baseline outcomes measurements similar;
4. baseline characteristics similar;
5. incomplete outcome data;
6. knowledge of the allocated interventions adequately prevented during the study;
7. protection against contamination;
8. selective outcome reporting;
9. other risks of bias;
10. intervention independent of other changes (interrupted time series studies only);
11. shape of the intervention effect if prespecified (interrupted time series studies only);
12. intervention unlikely to affect data collection (interrupted time series studies only).

We judged each potential source of bias as high, low, or unclear, and provided a quote from the study report together with a justification for our judgement in the Risk of bias in included studies table. We summarised the 'Risk of bias' judgements for each of the domains listed. We considered blinding separately for different key outcomes where necessary (e.g. for unblinded outcome assessment, risk of bias for all‐cause mortality may be very different than for a patient‐reported pain scale). Where information on risk of bias related to unpublished data or correspondence with a trialist, we noted this in the Risk of bias in included studies table. We did not exclude studies on the grounds of their risk of bias, but clearly reported the risk of bias when presenting the results of the studies.

When considering intervention effects, we took into account the risk of bias of the studies that contributed to that outcome.

We conducted the review according to this published protocol and have reported any deviations form it in the Differences between protocol and review section of this review.

##### Assessment of methodological limitations of included studies for the secondary objectives

For the secondary objectives, the included studies comprised a multitude of study designs and study aims, including case studies that were primarily descriptive. We were unable to find an accepted tool designed to appraise methodological limitations that could accommodate this variation in study design. We, therefore, piloted a newly developed tool for assessing the limitations of sources, such as programme reports, that do not use typical empirical research designs. One review author assessed the limitations of the studies using the Ways of Evaluating Important and Relevant Data (WEIRD) Tool (Lewin 2019) and a second review author checked the assessments. The tool, which is currently being piloted in EPOC and other systematic reviews, is available in Appendix 3.

For each item/question in the tool, the review author selected one of the following response options:

- Yes ‐ the item was addressed adequately in the source
- Unclear ‐ it is not clear if the item was addressed adequately in the source
- No ‐ the item was not addressed adequately in the source
- Not applicable ‐ the item is not relevant to the source being assessed

Based on the assessments for each WEIRD tool item, an overall assessment of the limitations of the source was made as follows:

- Where the assessments for most items in the tool were 'Yes' ‐ no or few limitations
- Where the assessments for most items in the tool were 'Yes' or 'Unclear' ‐ minor limitations
- Where the assessments for one or more questions in the tool were 'No' ‐ major limitations

The overall assessment for each source was then used as part of the GRADE‐CERQual assessment of how much confidence to place in each secondary objective finding.

#### Measures of treatment effect

For the analyses of the **primary objectives**, we reported means and proportions, where appropriate. When applicable, we estimated the effect of the intervention using risk ratio or risk difference for dichotomous data, together with the associated 95% confidence interval, and mean difference or standardised mean difference for continuous data, together with the associated 95% confidence interval. We ensured that an increase in scores for continuous outcomes could be interpreted in the same way for each outcome, explained the direction to the reader, and reported where the directions were reversed, if this was necessary.

#### Unit of analysis issues

For the analyses of the **primary objectives**, we performed data analysis at the same level as the allocation to avoid unit of analyses errors. We did not identify any cluster‐randomised trials for inclusion in the review. See Appendix 4 for methods specified in the protocol (Vasudevan 2019) but not used in the review.

#### Dealing with missing data

For the analyses of the **primary objectives**, we intended to contact investigators in order to verify key study characteristics and request missing outcome data (e.g. when a study was identified as abstract only), but this was not an issue.

#### Assessment of heterogeneity

For the analyses of the primary objectives, we intended to assess the heterogeneity of studies, but due to insufficient numbers of studies identified, we did not conduct the assessment. See Appendix 4 for methods specified in the protocol (Vasudevan 2019) but not used in the review.

#### Assessment of reporting biases

For the analyses of the **primary objectives**, we did not explore the impact of including studies with missing data since this was not an issue. See Appendix 4 for methods specified in the protocol (Vasudevan 2019) but not used in the review.

#### Data synthesis

For the analyses of the **primary objectives**, we proposed to undertake meta‐analyses only where this was meaningful, i.e. if the treatments, participants, and the underlying clinical question were similar enough for pooling to make sense. See Appendix 4 for methods specified in the protocol (Vasudevan 2019) but not used in the review.

To address the first of the **secondary objectives** (to describe the range of strategies used to implement birth‐death notification via mobile device), we presented the range of strategies that we identified in a table format.

To address the second of the **secondary objectives** (to identify factors influencing the implementation of birth‐death notification via mobile device), one review author familiarised themself with the extracted data and then applied the SURE framework, moving between the data and the themes covered in the framework, but also searching for additional themes until all the extracted data had been assessed. Two review authors then assessed, discussed and agreed upon the definitions and boundaries of each of the emerging themes.

To develop the implications for practice, one review author went through each finding, identified factors that may influence the implementation of the intervention, and developed prompts for future implementers. These prompts were reviewed by at least one other review author. These prompts are not intended to be recommendations, but are instead phrased as questions to help implementers consider the implications of the review findings in their context. The questions are presented in the ‘Implications for practice’ section.

#### Subgroup analysis and investigation of heterogeneity

If meaningful, we planned to carry out the following subgroup analyses:

1. by study setting (e.g. high‐income versus low‐ and middle‐income countries; urban versus rural);
2. by whether there was an existing CRVS (paper‐based) system in place versus no CRVS system in place at all;
3. by whether the notification was formal (i.e. for civil registration) versus informal (for purposes other than civil registration).

We proposed to use the following outcomes in subgroup analysis.

##### For birth notifications via mobile device

- coverage (e.g. proportion) of births notified via mobile device;
- timeliness of birth notifications via mobile device (e.g. time between birth and birth notification via mobile device);
- timeliness of receipt of newborn or child health services (e.g. immunisations) in response to birth notifications via mobile device (i.e. time between birth and receipt of services).

##### For death notifications via mobile device

- coverage (e.g. proportion) of deaths notified via mobile device;
- timeliness of death notifications via mobile device (i.e. time between death and death notification via mobile device);
- timeliness of cause of death ascertainment, reporting to a disease surveillance system, or both, in response to death notifications via mobile device (i.e. time between death and cause of death ascertainment).

#### Sensitivity analysis

See Appendix 4 for methods related to subgroup analysis and investigation of heterogeneity for the primary objectives that were specified in the protocol (Vasudevan 2019) but not used in the review.

#### Summary of findings and assessment of the certainty of the evidence

For the **primary objectives**, two review authors independently assessed the certainty of the evidence (high, moderate, low, and very low), using the five GRADE considerations (risk of bias, consistency of effect, imprecision, indirectness, and publication bias (Guyatt 2008)). We used methods and recommendations described in Section 8.5 and Chapter 12 of the *Cochrane Handbook for Systematic Reviews of interventions* (Higgins 2011), and the EPOC worksheets (EPOC 2017d), and GRADEpro software (GRADEpro GDT). We resolved disagreements on certainty ratings by discussion and have provided justification for decisions to down‐ or upgrade the ratings, using footnotes in the table. We used plain language statements to report these findings in the review (EPOC 2017e).

We summarised our findings in 'Summary of findings' tables (EPOC 2017d) for the main intervention comparisons, and included the most important outcomes and the certainty of the evidence for these outcomes.

For the **secondary objectives,** one review author used the GRADE‐CERQual (Confidence in the Evidence from Reviews of Qualitative research) approach to assess our confidence in each finding (Lewin 2018) and a second review author checked the assessments. GRADE‐CERQual assesses confidence in the evidence, based on the following four key components: methodological limitations of included studies; coherence of the review finding; adequacy of the data contributing to a review finding; and relevance of the included studies to the review question. After assessing each of the four components, we made a judgement about the overall confidence in the evidence supporting the review finding. We assessed confidence as high, moderate, low, or very low. The final assessment was based on consensus among the two review authors. All findings started as high confidence and were then graded down if there were important concerns regarding any of the GRADE‐CERQual components.

We presented summaries of the findings and our assessments of confidence in these findings in Table 2. We also presented detailed descriptions of our confidence assessment in Appendix 5.

## Results

### Description of studies

#### Results of the search

We included 21 studies in the review. We also found three ongoing studies and one study awaiting classification. Figure 2 summarises the study selection process as a PRISMA flowchart. For an overview of the included studies, see the Characteristics of included studies table. For an overview of the studies that we excluded during full‐text review, see the Characteristics of excluded studies table.

**2:**
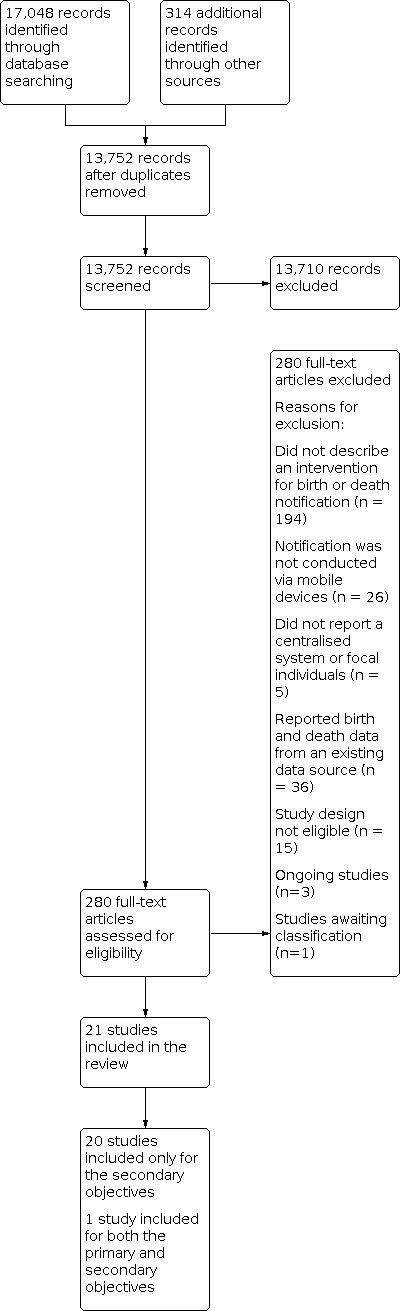
**Study flow diagram.**

From the 21 included studies, we identified one study that met the inclusion criteria for the **primary objectives**. This study described a birth notification intervention (Xeuatvongsa 2016). We did not find any studies that described a death notification intervention and that met the inclusion criteria for the primary objectives. We identified three ongoing studies that appeared to meet the inclusion criteria for the primary objectives and that are described in the Characteristics of ongoing studies table. The study awaiting classification is reported in the Characteristics of studies awaiting classification.

All 21 included studies addressed the **secondary objectives** (Andreatta 2011; ANISA 2016; eCRVS‐Mozambique 2017; Gisore 2012; MBRG 2014; MBRL 2011; MBRP 2015; MBRT 2016; Moshabela 2015; MOVE‐IT 2013; mSIMU 2017; mTika 2016; MVH 2017; mVRS 2017; Ngabo 2012; NIMDS 2019; Pascoe 2012; RapidSMS 2012; Van Dam 2015; Xeuatvongsa 2016; Yugi 2016).

#### Included studies

##### Study design and comparisons

The study addressing the **primary objectives** employed a controlled before‐after study design. (Xeuatvongsa 2016). The comparison was standard of care. This study measured the following outcomes: coverage and timeliness of birth notification, and coverage and timeliness of post‐notification health services.

Many studies addressing the **secondary objectives** were descriptive reports of programmes rather than formal qualitative or quantitative studies (Characteristics of included studies). The three studies that used rigorous study designs were controlled before‐after studies (mTika 2016; Xeuatvongsa 2016) and a cluster‐randomised trial (mSIMU 2017). One of these studies was also included in relation to the primary review objectives (Xeuatvongsa 2016), while the other two did not report the necessary outcomes for inclusion in relation to the primary review objectives. For all studies addressing the secondary objectives (including the before‐after studies and the randomised trial), most of the data we extracted were based on operational data. In many cases, the data were taken from the discussion section or other sections of the report, and were often based on the report authors’ own observations.

##### Setting

The study that addressed the **primary objectives** was conducted in Lao People’s Democratic Republic (Xeuatvongsa 2016).

The studies that addressed the **secondary objectives** were also conducted in low‐ or middle income settings. Five studies took place in Asia: Lao People’s Democratic Republic (Xeuatvongsa 2016), Bangladesh (mTika 2016), Pakistan (ANISA 2016; MBRP 2015; NIMDS 2019), and India (ANISA 2016). Fifteen studies took place in sub‐Saharan Africa: Kenya (Gisore 2012; mSIMU 2017), Mozambique (eCRVS‐Mozambique 2017), Tanzania (MBRT 2016; MOVE‐IT 2013; Pascoe 2012), Zambia (Van Dam 2015), Liberia (MBRL 2011), Ghana (Andreatta 2011; MBRG 2014), Uganda (mVRS 2017), Rwanda (Ngabo 2012), South Sudan (Yugi 2016), Nigeria (RapidSMS 2012) and Senegal (Moshabela 2015). One study took place in the Middle East: Syria (MVH 2017). There were no studies in high‐income settings.

With the exception of the eRegister platform (Van Dam 2015) in Lusaka, Zambia, all included studies focussed on identification of births and deaths in rural, remote, or marginalised populations who are typically under‐represented in civil registration processes or traditionally seen as having poor access to health services. The mTika study implemented a birth notification intervention in Dhaka, but focussed on populations in urban slums (mTika 2016).

##### Participants

We classified individuals providing notifications into one of four categories: lay health workers, family members, healthcare organisations, and community‐based informants.

In the study addressing the **primary objectives**, birth notification was conducted by healthcare workers and a cadre of lay health workers called village health workers (Xeuatvongsa 2016).

In most of the studies addressing the **secondary objectives**, notification of births and deaths was conducted by lay health workers.

- Lay health workers included community‐based and facility‐based workers such as traditional birth attendants, immunisation providers, community health workers (e.g. Lady Healthcare Worker, Accredited Social Health Activists), and village heath volunteers. (Andreatta 2011; MBRL 2011; MBRT 2016; Moshabela 2015; mTika 2016; Ngabo 2012; NIMDS 2019; RapidSMS 2012; Van Dam 2015; Xeuatvongsa 2016; Yugi 2016)
- In one of these studies, individuals from healthcare organisations and NGOs (non‐governmental organisations) that were part of the Syria Response Turkey Health Cluster used the Monitoring Violence against Health Care (MVH) tool to notify deaths (MVH 2017).
- Eight of these 21 studies included community‐based informants such as village elders, village chiefs, community volunteers, village reporters, marriage registrars, telecom agents, village executive officers, or village residents with access to a mobile phone (ANISA 2016; eCRVS‐Mozambique 2017; Gisore 2012; MBRG 2014; MBRP 2015; MOVE‐IT 2013; mSIMU 2017; mVRS 2017).
- In two of the 21 studies, mothers or other family members were provided instructions or resources to directly report births to a centralised server (ANISA 2016; mTika 2016)

##### Interventions for notification of births and deaths via mobile devices

The study addressing the **primary objectives** only implemented birth notification.

Among the 22 studies addressing the **secondary objectives**:

- Nine implemented birth notification only (ANISA 2016; MBRL 2011; MBRP 2015; MBRT 2016; mSIMU 2017; mTika 2016; mVRS 2017; RapidSMS 2012; Xeuatvongsa 2016).
- Five implemented death notification only (MVH 2017; NIMDS 2019; Pascoe 2012; Van Dam 2015; Yugi 2016).
- Seven implemented both birth and death notification via mobile devices (Andreatta 2011; eCRVS‐Mozambique 2017; Gisore 2012; MBRG 2014; Moshabela 2015; MOVE‐IT 2013; Ngabo 2012).

Eight studies described efforts to increase birth or death notification in conjunction with the national civil registration authority (eCRVS‐Mozambique 2017; MBRG 2014; MBRL 2011; MBRP 2015; MBRT 2016; MOVE‐IT 2013; mVRS 2017; RapidSMS 2012), while the remaining studies used birth or death notification to increase the coverage or timeliness of health services (Andreatta 2011; ANISA 2016; Gisore 2012; Moshabela 2015; mSIMU 2017; mTika 2016; Ngabo 2012; NIMDS 2019; Van Dam 2015; Xeuatvongsa 2016), and disease surveillance programs (Pascoe 2012; Yugi 2016). One study collected data on mortality resulting from attacks on healthcare organisations to assess violations of international humanitarian laws during war (MVH 2017).

The majority of the studies used basic mobile phones with voice and SMS capabilities. Birth notification was typically relayed as a text message (Andreatta 2011; MBRT 2016; MOVE‐IT 2013; mSIMU 2017; Ngabo 2012; mTika 2016; RapidSMS 2012), via phone call (ANISA 2016; Xeuatvongsa 2016), or via USSD (eCRVS‐Mozambique 2017; mVRS 2017). In one study in Kenya, each pair of village elder and registry administrator determined their modality of mobile phone communication (Gisore 2012). For birth notification, several studies used smartphone‐based apps. (MBRG 2014; MBRL 2011; MBRP 2015; MBRT 2016; Moshabela 2015) Most common modalities of death notification were SMS (Andreatta 2011; Ngabo 2012; NIMDS 2019; Yugi 2016) or smartphone‐based apps (Moshabela 2015; MVH 2017; Pascoe 2012; Van Dam 2015).

Some studies used open source data collection platforms such as RapidSMS (Ngabo 2012; RapidSMS 2012), Nokia Data Gathering (MBRL 2011), CommCare (Van Dam 2015), ChildCount+ (Moshabela 2015) and District Health Information Software 2 (DHIS2) (Pascoe 2012). System interoperability with national‐level health information systems was described poorly in the included sources. Only three studies described linkages of birth or death notification information to national‐level systems: DHIS2 in Tanzania (Pascoe 2012), a national Data Health Information System (DHIS) in South Sudan (Yugi 2016), and the Bangladesh Ministry of Health and Family Welfare’s Management Information System (mTika 2016).

##### Funding and conflicts

Sixteen studies listed their sources of funding (Andreatta 2011; ANISA 2016; Gisore 2012; MBRG 2014; MBRL 2011; MBRP 2015; MBRT 2016; MOVE‐IT 2013; mSIMU 2017; mTika 2016; MVH 2017; Ngabo 2012; RapidSMS 2012; Van Dam 2015; Xeuatvongsa 2016; Yugi 2016). Conflict of interest statements were available in reports of 12 studies (Andreatta 2011; ANISA 2016; Gisore 2012; MBRL 2011; Moshabela 2015; mTika 2016; MVH 2017; Ngabo 2012; NIMDS 2019; Van Dam 2015; Xeuatvongsa 2016; Yugi 2016). 

#### Excluded studies

We excluded 242 studies from the review following full‐text screening. Studies were excluded because they did not describe an intervention for birth or death notification (n = 160); notification was not conducted via mobile devices or the use of mobile devices for notification was poorly described (n = 26); they did not report a centralised system or focal individuals for birth or death notification (n = 5); the studies used existing sources of data (n = 36); or the publications were not of relevant design (n = 15) (see Characteristics of excluded studies).

### Risk of bias in included studies

#### Risk of bias in included studies addressing the primary objective

The study that met the eligibility criteria for addressing the primary objective (Xeuatvongsa 2016) used a controlled before‐after study design. We judged the study as having high or unclear risk across various criteria, as described in Table 3.

**1:** Risk of bias in the included study for the primary objective (Xeuatvongsa 2016)

**Bias**	**Authors' judgement^a^**	**Support for judgement**
Random sequence generation (selection bias)	High risk	Not a randomised controlled trial
Allocation concealment (selection bias)	High risk	Not a randomised controlled trial
Differences in baseline characteristics (selection bias)	High risk	(pg 5779) Participants different at baseline between intervention and control groups with respect to religion, ethnicity, maternal education, and HepB‐birth dose receipt
Baseline outcome measurements similar (selection bias)	Low risk	Similar outcomes measured at baseline
Blinding of participants and personnel (performance bias)	High risk	Blinding not possible
Blinding of outcome assessment (detection bias)	Unclear risk	Not reported
Incomplete outcome data (attrition bias)	High risk	Certain villages excluded due to lack of cold chain, large proportion of villages not visited. No details on the number of births registered, timeliness, only survey results from small proportion of HCWs
Selective reporting (reporting bias)	Unclear risk	Study protocol not available
Protection against contamination	High risk	Health workers in intervention and control groups using mobile phones
Other bias	Low risk	No other bias

^a^Judgement specifies whether there is a low, unclear, or high risk of bias.

#### Methodological limitations of included studies addressing the secondary objective

There was variation in the overall assessment of the limitations of the studies addressing the secondary objectives (Table 4).

**2:** Methodological limitations of the included studies for the secondary objectives ^a^

**Study ID**	**Clearly stated aim, objective or purpose?**	**Clear description of the source of the information (transparency)?**	**Clear description of the programme,intervention, policy or reform?**	**Clear description of the context/s?**	**Is the information accurate?**	**Is the evidence representative?**	**Any limitations of the information and/or methods discussed?**	**Is evidence provided to support any findings or conclusions made?**	**Relevant rights and ethics considerations described (empirical studies only)**	**Interests declared and any potential conflicts of interest noted?**	**Overall assessment^b^**	**Explanation of WEIRD assessment**
Andreatta 2011	Yes	Yes	Yes	Unclear ‐ limited details on context presented	Unclear ‐ some details of data analysis not presented	Unclear ‐ small sample size	Yes	Unclear ‐ limited evidence to support implementation outcomes	Yes	Yes ‐ authors have no conflicts to declare	**Minor limitations**	Concerns about details of the context, accuracy, representativeness of evidence, and evidence to support findings and conclusions made.
ANISA 2016	Yes	Yes	Unclear ‐ only some sub‐assessment criteria described	Unclear ‐ only some sub‐assessment criteria described	Unclear ‐ insufficient information to assess accuracy	Unclear ‐ insufficient information to assess representativeness	Unclear ‐ insufficient information to assess limitations	Unclear ‐ insufficient details on evidence to support findings.	Yes	Yes ‐ authors have no conflicts to declare	**Minor limitations**	Concerns about description of source information, context, accuracy of information, representativeness, limitations, and evidence to support conclusions.
eCRVS‐Mozambique 2017	Unclear ‐ stated aim does not include assessment of implementation factors	No ‐ No description of source of information	Yes	Yes	Unclear ‐ Source material does not describe efforts to ensure that the information is complete and accurate	Yes	Yes	No ‐ not a study	Not applicable	Not applicable	**Significant/major limitations**	Concerns about relevance of study aims, methods, study limitations or conflicts of interest information presented with respect to review objectives. Source material did not describe any efforts to ensure that the information presented was complete and reliable.
Gisore 2012	Yes	Yes	Yes	Yes	Yes	Yes	Yes	Yes	Yes	Yes ‐ authors have no conflicts to declare	**No or few limitations**	
MBRG 2014	Unclear ‐ Stated aim does not include assessment of implementation factors.	Unclear ‐ some sources are referenced but others potentially missed	Unclear ‐ only some sub‐assessment criteria described	Unclear ‐ only some sub‐assessment criteria described	Unclear ‐ Source material does not describe efforts to ensure that the information is complete and accurate	No ‐ not a study	No ‐ not described	No ‐ describes implementation but there is no associated evidence or empirical study	Not applicable	Not applicable	**Significant/major limitations**	Concerns about relevance of study aims, methods, study limitations and information presented with respect to review objectives. Source material did not describe any efforts to ensure that the information presented was complete and reliable.
MBRL 2011	Yes	Yes	Yes	Yes	Unclear ‐ Source material does not describe efforts to ensure that the information is complete and accurate	Yes	Yes	Yes	No ‐ not described	No‐not described	**Significant/major limitations**	Concerns about accuracy of evidence, ethical considerations, and reporting of conflicts of interest
MBRP 2015	Unclear ‐ Stated aim does not include the assessment of implementation factors	No ‐ not described	Yes	Yes	Unclear ‐ Source material does not describe efforts to ensure that the information is complete and accurate	No ‐ not an empirical study	Unclear ‐ some limitations described but others potentially missed.	No ‐ describes implementation but there is no associated evidence or empirical study	Not applicable	Not applicable	**Significant/major limitations**	Concerns about accuracy of source materials, study limitations and information presented with respect to review objectives. Source material did not describe any efforts to ensure that the information presented was complete and reliable.
MBRT 2016	Yes ‐ Purpose of the source material is not stated but can be derived as follows: To describe the implementation of a mobile birth registration programme in Tanzania	No ‐ Some quotes presented but no description of the source of information	Yes	Yes	Unclear ‐ Source material does not describe efforts to ensure that the information is complete and accurate	No ‐ not an empirical study	Unclear ‐ Lessons learned are presented but not phrased as limitations	No ‐ describes implementation but there is no associated evidence or empirical study	Not applicable	Not applicable	**Significant/major limitations**	Concerns about accuracy of evidence, its representativeness, and description of study limitations
Moshabela 2015	Yes	Yes	Yes	Yes	Yes	Unclear ‐ small sample size	Yes	Yes	Not applicable	Yes ‐ authors have no conflicts to declare	**Minor limitations**	Concerns about representativeness of evidence
MOVE‐IT 2013	Yes	Unclear ‐ sources of lessons learned not described	Yes	Yes	Yes	Unclear ‐ sources of lessons learned not described	Unclear ‐ sources of lessons learned not described	Unclear ‐ sources of lessons learned not described	Not applicable	Unclear ‐ funding source described but no other conflicts declared.	**Minor limitations**	Concerns about description of source of information, representativeness of evidence, limitations, declarations of conflict of interest, and evidence related to findings
mSIMU 2017	Yes	Yes	Yes	Yes	Yes	Yes	Yes	Yes	Unclear ‐ not described	Unclear ‐ funding source listed but no conflict of interest statement available	**Minor limitations**	Lack of information on ethical considerations and conflict of interest declaration
mTika 2016	Yes	Yes	Yes	Yes	Unclear ‐ Details of the qualitative data analysis are sparse	Yes	Unclear ‐ Limitations of qualitative interviews not discussed	Unclear ‐ No quotes or underlying evidence presented for qualitative interviews	Yes	Yes ‐ authors have no conflict of interest to declare	**Minor limitations**	Concerns about accuracy of the evidence, evidence to support findings and description of study limitations.
MVH 2017	Yes	Yes	Yes	Yes	Yes	Yes	Yes	Yes	Unclear ‐ not described	Yes ‐ authors have no conflicts to declare	**No or few limitations**	
mVRS 2017	Yes	Yes	Yes	Yes	Yes	Yes	Yes	Yes	Yes	Unclear ‐ insufficient information to assess whether ethical approval was necessary in the study settings.	**No or few limitations**	
Ngabo 2012	Yes	Yes	Yes	Yes	Unclear ‐ only some sub‐assessment criteria described	Yes	Yes	Yes	Not applicable	Yes ‐ authors have no conflicts to declare	**No or few limitations**	
NIMDS 2019	Yes	Yes	Yes	Yes	Yes	Yes	Yes	Yes	No‐Not described	Yes ‐ authors have no conflicts to declare	**No or few limitations**	
Pascoe 2012	Yes	Yes	Yes	Yes	Unclear ‐ only some sub‐assessment criteria described	Unclear ‐ small sample size	Yes	Yes	No ‐ not described	No ‐ not described	**Significant/major limitations**	Concerns about accuracy of evidence, small sample size, lack of conflict of interest disclosure and ethical considerations
RapidSMS 2012	Yes ‐ Purpose of the source material is not stated but can be derived as follows: To describe the implementation of the RapidSMS programme for birth registration in Nigeria	No ‐ not described	Unclear ‐ Some assessment sub‐criteria are not fully described. There is no description of the materials used in the programme, infrastructure and resources required, or mechanisms to ensure that the programme was implemented as intended.	Unclear ‐ Some assessment sub‐criteria are not fully described. There is no description of the historical, sociocultural, socioeconomic or ethical context, the political, legal, governance, policy context, including relevant key events or policy initiatives, or a clear description of how different stakeholders were involved in the programme.	Unclear ‐ Source material does not describe efforts to ensure that the information is complete and accurate	Unclear ‐ The description is not based on a sampling approach and there is no rationale or description of how generalizations to wider populations or settings were made.	No ‐ Not described	No ‐ not described	Not applicable	Not applicable	**Significant/major limitations**	Concerns about the descriptions of transparency, methods, accuracy of evidence, study limitations presented in the source materials
Van Dam 2015	Yes	Yes	Yes	Unclear ‐ only some sub‐assessment criteria described	Yes	Unclear ‐ small sample size	Yes	Yes	Not applicable	Yes ‐ author affiliations which may be perceived as conflict of interest are disclosed	**Minor limitations**	Concerns about description of context, accuracy of information, and representativeness of evidence
Xeuatvongsa 2016	Yes	Yes	Yes	Yes	Yes	Unclear ‐ small sample size	Yes	Yes	Yes	Yes ‐ authors have no conflicts to declare	**Minor limitations**	Concerns about representativeness of evidence
Yugi 2016	Yes	Yes	Yes	Yes	Yes	Unclear ‐ small sample size	Yes	Yes	Not applicable	Yes ‐ authors have no conflicts to declare	**Minor limitations**	Concerns about description of source of information, representativeness and evidence related to findings

^a^Details of the WEIRD tool assessment criteria and prompts are available in Appendix 3. ^b^*No or few limitations*: when the answer to most questions in the tool is YES *Minor limitations*: when the answer to most questions in the tool is YES or UNCLEAR *Significant / major limitations*: when the answer to one or more questions in the tool is NO

- Five studies were assessed as having no or few limitations (Gisore 2012; MVH 2017; mVRS 2017; Ngabo 2012; NIMDS 2019)
- Nine studies were assessed as having minor limitations (Andreatta 2011; ANISA 2016; Moshabela 2015; MOVE‐IT 2013; mSIMU 2017; mTika 2016; Van Dam 2015; Xeuatvongsa 2016; Yugi 2016)
- Seven studies were assessed as having significant/major limitations (eCRVS‐Mozambique 2017; MBRG 2014; MBRL 2011; MBRP 2015; MBRT 2016; Pascoe 2012; RapidSMS 2012)

### Effects of interventions

See: Table 1; Table 2

#### Primary objective: *Effect of birth‐death notification by mobile device*

##### Comparison 1: Birth notification via mobile devices compared with standard practice

One controlled before‐after study was included in this comparison (Xeuatvongsa 2016). This study aimed to improve the coverage of postnatal home visits within 24 hours of birth, specifically for provision of the birth dose of the Hepatitis B vaccination. In this study, Village Health Volunteers (VHVs) used mobile phones to communicate with Health Care Workers (HCWs) and notify them of impending deliveries as well as births. During study implementation, the VHVs and HCWs in the intervention sites, but not the control sites, received mobile phones and mobile phone credit.

###### 1.1 Coverage of births notified via mobile devices

The study assessed the proportion of VHVs who reported notifying a HCW about deliveries or births using mobile phones and the proportion of HCWs who reported receiving a notification from VHV about deliveries or birth using mobile phones. We are uncertain if birth notification via mobile device improves the coverage of birth notification as the certainty of the evidence was very low (Table 1).

###### 1.2 Timeliness of birth notification via mobile devices

The study assessed the proportion of VHVs who reported notifying HCWs either during labor or within one day of birth using mobile phones and the proportion of HCWs who reported receiving a notification from VHV about imminent deliveries or within one day of birth using mobile phones. We are uncertain if birth notification via mobile device improves the timeliness of birth notification as the certainty of the evidence was very low (Table 1).

###### 1.3 Legal birth registrations in response to birth notifications via mobile device

The study did not assess this outcome.

###### 1.4 Timeliness of legal birth registrations in response to birth notification via mobile device

The study did not assess this outcome.

###### 1.5 Coverage of newborn or child health services in response to birth notification via mobile device

The study assessed the proportion of births where HCWs made postnatal care visits to homes and the proportion of births for which a Hepatitis B birth dose vaccination was provided within 30 days. We are uncertain if birth notification via mobile device improves coverage of newborn or child health services as the certainty of the evidence was very low (Table 1).

###### 1.6 Timeliness of receipt of newborn or child health services in response to birth notification via mobile device

The study assessed the proportion of births where Hepatitis B birth dose vaccination was administered within zero to one days; the proportion of births where Hepatitis B birth dose vaccination was administered within two to seven days; and the proportion of births where the HCW made a postnatal care home visit with 24 hours of notification. We are uncertain whether birth notification via mobile device improves the timeliness of or receipt of newborn or child health services as the certainty of the evidence was very low (Table 1).

##### Comparison 2: Death notification via mobile devices compared with standard practice

No studies were included that addressed this comparison.

#### Secondary objectives: Strategies used to implement birth and death notification via mobile devices, and factors that influence this implementation

##### 1. Strategies used to implement birth and death notification via mobile devices

For an overview of the strategies that were used in the included studies to implement birth‐death notification via mobile device, please see Table 5.

**3:** Strategies used to implement birth and death notification via mobile devices

**Study + setting**	**Notifier**	**Description of strategy**
MBRT 2016 Tanzania (Mbeya and Mwanza regions)	Health workers in government clinics	**Birth notification:** Health workers enter birth information using a mobile phone, either via a smartphone app interface or via SMS prompts on a basic phone. Data are transferred via an SMS protocol to a central database in the Registration, Insolvency and Trusteeship agency (RITA) Once the server at RITA returns a confirmation that the birth information is received, a birth certificate for the child is issued on the spot.
MBRP 2015 Pakistan (Panjab and Sindh provinces)	Community 'gatekeepers' (marriage registrars, lady health workers, and Telenor (telecom) agents)	**Birth notification:** Gatekeepers enter birth information using an android app, and capture images of any supporting documents using the phone’s camera. Data are transferred via internet or mobile USB to the Union Council (UC) secretary, who is responsible for civil registration records for residents of the union. The UC secretary checks details of information received on a tablet device and creates a unique birth record in the paper‐based UC register and the National Database and Registration Authority (NADRA) database. A confirmation SMS is sent upon registration to the parents. Birth certificate is issued after completion of formalities at the UC.
MBRG 2014 Ghana	Community volunteers	**Birth and death notification:** Community volunteers use android app to collect child’s details (name, gender, date of birth, other family details), and send data to a central database managed by the Ghana Births and Deaths Registry. Data are stored and an automated response is sent to the Births and Deaths Registry official in the field to issue a certificate for the child.
mSIMU 2017 Kenya (Nyanza province)	Village reporters working with the Health and Demographic Surveillance System (HDSS) programme	**Birth notification:** Village reporters send birth notification via SMS to Rapid‐SMS server. Server notifies field‐based community workers to screen and enrol infants in m‐SIMU study.
Gisore 2012 Kenya (Western Province)	Village Elders; registry administrator	**Birth and death notification:** Village elders use mobile phones to notify registry administrator of birth outcomes (including stillbirths and early neonatal deaths), and birth weight within 7 days post‐delivery. Village elder and registry administrator determine modality of mobile phone communication.
Van Dam 2015 Zambia (Lusaka)	Health workers	**Death notification:** Health workers used the eRegister system created using CommCare and deployed on Samsung Galaxy 2 tablet devices to enter information related to date and cause of death for Rheumatic Heart Disease patients.
ANISA 2016 Pakistan (Matiari district) and India	Families of pregnant women, Lady Health Workers (LHWs), Traditional Birth attendants (TBAs), or residents of the village with mobile phone access	**Birth notification:** Prepaid phone cards worth 100 Pakistani Rupees provided to families of pregnant women, LHWs, and TBAs for birth notification. In areas with no LHWs/TBAs, residents of the village reimbursed for phone calls to study staff for notifying births.
mTika 2016 Bangladesh	Mothers	**Birth notification:** During pregnancy, mothers receive unique code and instructions on sending SMS with birth details to the mTika server.
Xeuatvongsa 2016 Lao People’s Democratic Republic (Luang and Xayabuly provinces)	Village Health Workers (VHVs); Health Care Workers (HCWs)	**Birth notification:** VHVs and HCWs in intervention areas provided with mobile phones and airtime. VHVs notify HCWs of imminent deliveries and new births to trigger postnatal care home visits and HepB birth dose vaccination by HCWs.
Pascoe 2012 Tanzania (Pwani region)	Health workers	**Death notification:** Health workers use the District Health Information System2 (DHIS2) mobile app for weekly reporting of cases and deaths per the WHO Integrated Disease Surveillance and Response (ISDR) strategy.
Yugi 2016 South Sudan (Eastern Equatoria state)	Health facility staff, county Health Department staff	**Death notification:** Health facility staff use personal phones to send SMS to a county health department Android phone for weekly reporting of cases and deaths as per the WHO Integrated Disease Surveillance and Response (ISDR) strategy. Data from the Android phone are submitted to the national Data Health Information System (DHIS) via an interface that decodes the SMS data.
Andreatta 2011 Ghana (Sene district)	Birth attendants	**Death notification:** For each delivery attended, birth attendants use a predefined SMS protocol to send data on maternal demographics, post‐partum haemorrhage status, maternal death outcome, neonatal death outcome, and prenatal service delivery statistics. The SMS is sent to a central study phone, and the data are later transferred to a database.
Ngabo 2012 Rwanda (Northern province)	Community Health Workers	**Birth and death notification:** CHWs use RapidSMS system to report pregnancy outcomes including maternal and child deaths.
Moshabela 2015 Senegal (Northwest)	Community Health Workers	**Birth and death notification:** CHWs use Childcare+ to report births and cases of deaths of children under five, and women ages 12‐49 years.
MOVE‐IT 2013 Tanzania (Rufiji district)	Village Executive Officers (VEOs)	**Birth and death notification:** VEOs record birth or death information in facility registers and use mobile phones to send the information as a structured SMS to a central database linked to the district civil registry. VEOs provide copy of notification form to household members. Household members visit the District Civil Registrar’s office to complete birth or death registration, pay the fee, and collect the birth or death certificate.
eCRVS‐Mozambique 2017 Mozambique	Village chiefs	**Birth and death notification:** Village chiefs use USSD via mobile phone to notify the national e‐civil registration and vital statistics system regarding births or child deaths in their village. In response to the notification, the village chief receives a personal number (single citizen’s identification number) for the child. Families receive SMS when the birth or death certificate is ready.
MBRL 2011 Liberia (Bomi county)	Health workers	**Birth notification:** Health workers use the Nokia Data Gathering software to enter data related to births and send to a centralised server.
mVRS 2017 Uganda	Village chiefs	**Birth notification:** Birth notification is issued by the hospital administration or community notifier via mobile phone (USSD) to the Mobile Vital Registration System (mVRS). Upon online verification by the National Identification and Registration Authority (NIRA), the notifier is able to print, sign and issue the notification to parents or other family members.
MVH 2017 Syria, Turkey	Staff at all Turkey health cluster organisations (internal partners)	**Death notification:** An internal or external partner posts information to a 293‐member WhatsApp group. Members with physically verified information (via site visit or presence during incident) complete anonymous and confidential online alert form. Form includes location, attack type, facility type, extent of damage, who is affected, injuries and deaths. Triangulated, key data (location, type of service, modality of attack, deaths, and casualties) from forms distributed within 24 hours to all partners and donors.
RapidSMS 2012 Nigeria	Registrars	**Birth notification:** Registrars with unique identification numbers send birth information using SMS to the RapidSMS server.
NIMDS 2019 Pakistan (Punjab district)	Lady Health Supervisors (LHSs)	**Death notification:** Neonatal Infant and Maternal Deaths E‐surveillance System (NIMDS) Lady Health Workers (LHWs) and community midwives (CMWs) report neonatal, infant, or maternal deaths that have occurred by informing the respective Lady Health Supervisors (LHSs). LHS confirms death and sends a SMS with complete neonatal, infant, or maternal death string to the system from her registered mobile number. If the SMS reporting format is correct, then the system sends an auto confirmation message with a unique Case Number of that death. If the SMS reporting format is incorrect, the system generates an error message with an auto reply to the sender about the specific error string.

ASHA: Accredited Social Health Activists CHW: Community Health Worker CMW: Community Midwife DHIS2: District Health Information System2 (DHIS2)  HCW: Health Care Worker HDSS: Health and Demographic Surveillance System ISDR: Integrated Disease Surveillance and Response (ISDR) strategy LHS: Lady Health Supervisor LHW: Lady Health Worker mVRS: mobile Vital registration System NADRA: National Database and Registration Authority NIMDS: Neonatal Infant and Maternal Deaths E‐surveillance System NIRA: National Identification and Registration Authority RITA: Registration, Insolvency and Trusteeship agency TBA: Traditional Birth Attendant SMS: Short Message Service UC: Union Council USB: Universal Serial Bus USSD: Unstructured Supplementary Service Data VEO: Village Executive Officer  VHV: Village Health Volunteer WHO: World Health Organization

##### 2. Factors that influence the implementation of birth and death notification via mobile devices.

The 21 studies that addressed the secondary objectives described a variety of factors that could influence the implementation of birth and death notification using mobile phones. As described above, these findings are primarily based on the report authors’ own comments and observations and are not based on a formal data gathering or analysis process. Using the SURE framework as our starting point, we have grouped these findings as follows:

- Factors related to health system constraints in the implementation of birth and death notification via mobile devices;
- Factors related to characteristics of individuals providing birth and death notification via mobile devices;
- Factors related to characteristics of families for whom birth and death is notified via mobile devices;
- Factors related to characteristics of other stakeholders involved in birth and death notification via mobile devices;
- Factors related to the mobile technologies used for birth and death notification – this component is not in the original SURE framework but was added for the purposes of this review.

The SURE Framework also includes factors tied to social and political constraints. However, only a few studies described factors influencing the implementation of birth and death notification strategies using mobile phones that could be categorised as such. The summary of findings on factors that influence the implementation of birth and death notification via mobile devices are presented in Table 2.

###### A. Health system constraints in the implementation of birth and death notification via mobile devices

***Finding A.1. Geographic barriers hamper timeliness of birth and death notification conducted via mobile devices, as well as post‐notification services or processes (e.g. certification of birth or death) (moderate‐confidence finding).***

Digital devices could allow healthcare providers to more efficiently notify authorities about births and deaths. However, healthcare providers carrying mobile devices still need to reach families to ascertain that a birth has occurred and to gather information needed for the purposes of notification. Study authors described how geographical barriers hindered providers’ ability to reach families and gather the necessary information for notification and to deliver post‐notification services. In particular, two studies reported that accessibility challenges delay birth notification, which could then lead to delays in providing healthcare services that are due within 24 hours after birth (ANISA 2016; Xeuatvongsa 2016).

Primary challenges faced by providers in accessing families for birth or death notification by mobile phone or for the delivery of post‐notification services, were distance (ANISA 2016; Xeuatvongsa 2016), seasonal impassability of roads (Pascoe 2012), and lack of reliable and inexpensive transportation options (MOVE‐IT 2013; Pascoe 2012). In Tanzania, Pascoe and colleagues noted that during the rainy season, some roads were impassable by motor vehicles affecting the ability of health workers to travel there (Pascoe 2012).

Suggestions or efforts to improve accessibility to families for the purpose of timely notification via mobile devices or delivery of services centred on reducing the 'distance' between the health workers and the communities. In one study, staff were stationed at the project office to receive birth notification calls, and families and community health workers received prepaid phone cards to notify the project office of new births (ANISA 2016).

While health workers in some of the studies travelled to the family for the birth or death notification, families were usually expected to travel to facilities or the registrar’s office to access post‐notification services. Three studies described challenges faced by families in accessing post‐notification services such as certification of births or access to health services. For instance, transportation barriers impacted family members’ ability to access the registrar’s office to certify births and deaths and, in the case of births, to access health facilities to access post‐notification services (MBRT 2016; MOVE‐IT 2013; Ngabo 2012). For instance, in the MOVE‐IT project, transportation barriers and associated costs were cited as reasons for non‐certification of birth or death events by family members, following timely notification by health workers using mobile phones (MOVE‐IT 2013).

The studies made several suggestions on improving family members’ accessibility to registration services including increasing the number and proximity of ‘service points’ where registration can occur, and using digital systems for faster processing of registration information at these service points (ANISA 2016; MBRT 2016; mVRS 2017). Integration of birth registration with immunisation campaigns or other neonatal health services was suggested as one way to improve birth registration rates. Printing of birth certificates at the registration service points was also noted as a way of reducing the number of steps needed to complete registration, and for helping parents to avoid the ‘long expensive journey to a far‐away registration centre' (MBRT 2016).

Health workers using mobile devices for birth–death notification may also require supervision. But studies also reported challenges with in‐person supervision when access to communities was problematic. To resolve this issue, two studies reported using an online dashboard, which allowed officials/supervisors to remotely monitor birth notification data collected using mobile phones (MOVE‐IT 2013; Ngabo 2012).

***Finding A.2. Birth and death data collected using mobile devices can help health and civil registration systems identify problems and introduce appropriate quality improvements (low‐confidence finding).***

In several studies, authors described the benefits of collecting birth and death data via mobile phones as this could help identify problems, which again could lead to improvements in the organisation of healthcare services (MBRT 2016; Moshabela 2015; MVH 2017; NIMDS 2019; RapidSMS 2012). For instance, in the Millennium Villages Project in Senegal, study authors reported that verbal autopsy data collected via mobile phones during routine death surveillance enabled the early identification of increased maternal mortality rates in the region (Moshabela 2015). This, in turn, provided justification for implementing responsive quality improvement measures at the local hospital to reduce maternal deaths. A study in Pakistan described a maternal and neonatal death registration system with the purpose of identifying regions with high mortality rates and generating appropriate strategies to reduce mortality (NIMDS 2019). In studies in Tanzania and Nigeria, authors speculated that government accountability towards registration services may increase due to the real‐time availability of birth data notified from health facilities (MBRT 2016; RapidSMS 2012). In Nigeria, the availability of real‐time information was seen as a way of identifying poor‐performing (“lazy”) registrars (officials who validate, register and certify life events such as births and deaths) (RapidSMS 2012).

One study described how a WhatsApp‐based reporting tool was used in war zones to document violence against healthcare services and mortality among healthcare workers (MVH 2017). This data was used to document breaches of international humanitarian law protecting healthcare workers. These data were disseminated via monthly reports, infographics and advocacy. However, the authors noted that attacks on healthcare facilities and healthcare workers continued, despite the availability of real‐time data.

***Finding A.3. Health workers who lack familiarity with, or prior experience in, using mobile technologies may need rigorous training as well as post‐training support (moderate‐confidence finding).***

Most studies reported training health workers and community‐based notifiers prior to the implementation of birth and death notification via mobile devices (Andreatta 2011; Gisore 2012; MBRL 2011; MBRT 2016; MOVE‐IT 2013; mSIMU 2017; mTika 2016; Ngabo 2012; Van Dam 2015; Xeuatvongsa 2016; Yugi 2016). Studies reported that notifiers sometimes lacked familiarity with mobile devices or features prior to training; were unfamiliar with the digital communication protocols selected for delivering the notification (e.g. the format for SMS); or had gaps in clinical training, including aspects of health service delivery or disease aetiology; and health management. (Andreatta 2011; Gisore 2012; NIMDS 2019; Xeuatvongsa 2016; Yugi 2016). Some studies also reported that health workers faced technical challenges in learning to use phones for data collection, for instance making mistakes in composing the SMS notification string (mSIMU 2017; mTika 2016; NIMDS 2019).

Health workers’ knowledge gaps were mitigated by implementing rigorous training on the use of mobile technologies and the use of various communication formats for providing notification. Training sessions described in the included studies varied from day‐long workshops to multiple workshops lasting several days (Andreatta 2011; Gisore 2012; MBRL 2011; MOVE‐IT 2013; mSIMU 2017; Ngabo 2012; Xeuatvongsa 2016; Yugi 2016). Most studies reported using group training formats which typically involved interactive exercises and practice on using mobile phones to notify birth or death information (Andreatta 2011; Gisore 2012; MBRL 2011; mSIMU 2017; Ngabo 2012; Yugi 2016). For instance, in the M‐SIMU project in Kenya, village reporters were trained in groups of 30, followed by one‐on‐one training by field supervisors for those with persistent challenges in data entry (mSIMU 2017). Training materials included pictographic instructions and reference cards, and were typically translated into local languages (Andreatta 2011; mSIMU 2017; Ngabo 2012). In one study in Rwanda, national trainers collaborated on the development of training material development (Ngabo 2012).

Studies also described various strategies to address technical challenges in the use of mobile devices for notification. In Pakistan, authors reported simplifying the death notification SMS string by reducing its length and removing case‐sensitive text, leading to fewer errors in spelling, format, and string order (NIMDS 2019). In Kenya, health workers began data collection using phones in advance of the evaluation period so that initial technical issues with the use of phones for data collection could be rectified (mSIMU 2017). In other studies, health workers were given ad hoc post‐training support, mechanisms were established so that they could report problems and receive help, and continuous monitoring of data quality and timeliness was conducted (MBRT 2016; MOVE‐IT 2013; mTika 2016; Van Dam 2015; Yugi 2016). In one study in Zambia, the authors hypothesized that training needs for health workers may reduce in the future as the use of mobile devices becomes more pervasive (Van Dam 2015).

***Finding A.4. Local capacity to train future cadres of notifiers may be strengthened through 'train the trainer' approaches (low‐confidence finding).***

Two studies described approaches for local capacity building of trainers for training future cadres of health workers involved in birth and death notification using mobile devices (MBRL 2011; Ngabo 2012). One study reported a cascade training approach in Rwanda where, in the first stage, ten national trainers from the Ministry of Health were trained. The second stage involved training of district‐level supervisors and data managers by the national trainers and the final stage was training of over 400 community health workers by the district‐level supervisors (Ngabo 2012). A second study described including representatives from local IT companies in the pool of trainers to support the Liberian Ministry of Health and Social Welfare in future training sessions (MBRL 2011).

***Finding A.5. Mechanisms for continuous monitoring and supportive supervision are important for ensuring the quality and timeliness of birth and death data collected via mobile devices (moderate‐confidence finding).***

A number of studies noted that while rigorous training of health workers on how to conduct mobile device‐based data collection was critical for implementation, additional and continuous monitoring mechanisms were also crucial for ensuring data quality and timeliness (Andreatta 2011; MOVE‐IT 2013; mTika 2016; Ngabo 2012; Yugi 2016). A study in Ghana recommended cross‐verification of data for accuracy since favourable outcomes may be over‐reported while unfavourable ones are under‐reported by data collectors (Andreatta 2011). One study in Tanzania used simple feedback messages to acknowledge receipt of weekly disease surveillance reports or to remind health workers without timely submissions (Pascoe 2012). Uddin and colleagues were less specific, but cited the need for continuous monitoring of field activities and inclusion of project and technical staff input in order to mitigate implementation challenges for birth and death notification via mobile devices (mTika 2016).

One study described that quality assurance may be facilitated through the use of web‐based dashboards that track data (Ngabo 2012). In this study, authors reported that notifiers made fewer errors in data transmission over time (Ngabo 2012). In other studies, the authors suggested that supervision may be targeted to low‐performers, who could be identified rapidly through the availability of ‘real time’ digital performance data. For instance, the supervisory team from the MOVE‐IT project conducted spot checks on whether SMS messages were formatted correctly in addition to focussing on notifiers who had submitted data on no or few events prior to the supervision (MOVE‐IT 2013). One study described the use of compulsory reporting of reasons for failure to register newborns within 24 hours of birth as the basis for increased accountability and performance of study teams (ANISA 2016). Another study in South Sudan further highlighted the case for continuous monitoring – here the timeliness of reporting of disease surveillance data dropped in a county where the surveillance officer vacated his post (Yugi 2016). To mitigate such issues, the authors recommended identifying backup surveillance officers, in addition to quarterly review of performance with health workers (Yugi 2016).

***Finding A.6. Inadequate attention is paid to legal frameworks governing civil registration. These may need to be modified to allow notification via mobile device and the inclusion of new cadres of notifiers (low‐confidence finding).***

Three included studies discussed legal frameworks governing civil registration, and the limitations they may impose on the authority of the notifier to provide the full range of civil registration services (eCRVS‐Mozambique 2017; MBRP 2015; mVRS 2017). One study in Uganda reported that hospital administrators or community notifiers using the Mobile Vital Registration System (MVRS) were able to issue a lawful notification but did not have the authority to issue the birth certificate (mVRS 2017). Alternatives to legal reform (e.g. the use of a memorandum of understanding) were viewed as temporary fixes to allow the use of mobile devices in the notification process (MBRP 2015). Instead, authors recommended working with the government to incorporate modern methods of birth registration (including notification) in the law. In Mozambique (eCRVS‐Mozambique 2017), legal and policy reforms undertaken by the government to accommodate notification of births and deaths via mobile devices, included:

- Identification of acceptable technologies needed for birth and death notification via mobile devices;
- Acceptance of digital notifications over paper‐based notifications; and
- Development of systems used to uniquely identify individuals in the digital civil registration database, and updates to reflect how these processes might change due to the use of mobile devices for notification.

***Finding. A.7. The availability of adequate human resources to conduct birth and death notification via mobile devices may be facilitated by hiring new cadres of notifiers or recruiting existing cadres of health workers to undertake notification (moderate‐confidence finding).***

Studies reported the need for adequate numbers of trained, local staff to conduct birth and death notification. A lack of staff was seen as a potential constraint to scaling up birth and death notification strategies using digital devices. (Andreatta 2011; ANISA 2016; eCRVS‐Mozambique 2017; Gisore 2012; MBRL 2011; MBRT 2016; mVRS 2017; Pascoe 2012). Several studies suggested recruiting existing cadres of health workers not previously involved in notification (Andreatta 2011; ANISA 2016; eCRVS‐Mozambique 2017; MBRT 2016). In other studies, health workers and community leaders already engaged in paper‐based notification of births and deaths were equipped with mobile phones (ANISA 2016; Gisore 2012; Xeuatvongsa 2016). For instance, in Kenya, village leaders already responsible for recording home births and deaths were equipped with mobile phones for notification of vital data and birth weights (Gisore 2012). Another study in Tanzania suggested that relieving health workers of administrative tasks may allow them to direct more effort into notification and other health services (Pascoe 2012). Several studies reported hiring additional staff in supervisory and project coordination roles to support and monitor individuals providing vital notification via mobile devices (ANISA 2016; MOVE‐IT 2013; Yugi 2016).

***Finding A.8. Implementing birth and death notification via mobile devices may be influenced by underlying health and civil registration system infrastructure, resources, and processes (low‐confidence finding).***

In addition to adequate numbers of notifiers, several studies pointed to the need for strong underlying health and civil registration systems when mobile phones are used for birth and death notification (ANISA 2016; Gisore 2012; MBRL 2011; Moshabela 2015; MOVE‐IT 2013; mVRS 2017; Ngabo 2012). In those studies, birth and death data were collected as part of routine study activities, prior to implementation of mobile devices for collection of this data (Gisore 2012; Moshabela 2015; Ngabo 2012). For instance, one study in Rwanda described “an already existing and well organised community based health programme, the PBF approach coupled with the scale‐up of community health insurance…and perfect delineation of administrative boundaries with clearly defined roles and responsibilities for CHWs…” as reasons for successful implementation of birth and death notification via mobile phones (Ngabo 2012). A study in Liberia described the need to establish necessary technical infrastructure and training of staff for the implementation of their mobile birth registration system (MBRL 2011). In rural areas in Syria, the lack of more than one partner health organisation made it challenging to verify accounts of death following an attack from independent sources (MVH 2017).

###### B. Factors related to individuals providing birth and death notification via mobile devices

***Finding B.1. Costs incurred by health workers sending notifications using mobile personal phones may need to be reimbursed to facilitate sustained use of these technologies for notification (moderate‐confidence finding).***

Some studies discussed the need to cover the costs incurred by notifiers when using their own phones to notify births and deaths (mSIMU 2017; Ngabo 2012; Pascoe 2012; Xeuatvongsa 2016; Yugi 2016). In a study in Lao PDR, health workers in the intervention and control arms used their mobile phones for notification. While health workers in the intervention arm were compensated for the use of mobile phone credit, those in the control arm were not. In the absence of compensation, the lack of phone credit was reported more frequently in the control arm as the reason for not being able to use the phone for notification (Xeuatvongsa 2016).

To address costs incurred by notifiers when using their own phones, most studies reported providing phone credit to the notifiers (mSIMU 2017; Pascoe 2012; Xeuatvongsa 2016; Yugi 2016). A study in Rwanda reported the use of a reverse billing system through which the Rwandan government covered the costs of phone use by the health workers (Ngabo 2012). Another study in Kenya reported that the Village Elders were asked to purchase the airtime for their phones themselves (Gisore 2012). Further studies are needed to describe the impact of costs incurred by the notifiers on the timeliness or coverage of notification.

***Finding B.2. The use of mobile phones for notification is acceptable to health workers, and helps them to undertake their job responsibilities (moderate‐confidence finding).***

Several studies reported high acceptability among health workers for using mobile devices to conduct notification (mSIMU 2017; Ngabo 2012; NIMDS 2019; Pascoe 2012; Van Dam 2015; Yugi 2016). These studies found that health workers using mobile phones for notification:

- Displayed more self‐confidence or reported being more proactive in finding and reporting pregnancies due to reminders sent to their phones (Ngabo 2012; NIMDS 2019);
- Spent less time than during the pilot study period in composing and sending notification (NIMDS 2019);
- Reported spending more time delivering services rather than reporting data, especially when reports were submitted electronically rather than in‐person (Pascoe 2012);
- Reported earning more trust and respect from families due to their ability to communicate with and coordinate emergency services with health facilities (Ngabo 2012);
- Reported that the phone‐based notification system was easy to use (mSIMU 2017; NIMDS 2019; Van Dam 2015; Yugi 2016).

Strategies reported in the studies to improve intervention acceptability and adoption among health workers included:

- Using an iterative, human‐centred process for the development of the mobile application or format for communication (NIMDS 2019);
- Providing financial incentives based on performance (see Finding B.3.).

***Finding B.3. Health workers’ adoption of mobile birth and death notification strategies may be affected by competing priorities and the availability of adequate incentives (moderate‐confidence finding).***

Several studies reported challenges with the successful adoption of strategies for mobile birth and death notification by health workers, and this was seen to be due to competing priorities and a lack of adequate incentive structures (MOVE‐IT 2013; mSIMU 2017; mTika 2016; MVH 2017). The MOVE‐IT project in Tanzania reported that some Village Executive Officers did not follow up proactively to report new births, sometimes even waiting for the parents or relatives of the newborn to come to their offices to initiate the process (MOVE‐IT 2013). Reasons provided for this lack of engagement included the busy schedules of the Village Executive Officers; home visits not being part of routine job responsibilities; and a lack of incentives or commissions for reporting births. Similarly, a study in Kenya suggested that the small incentive payment provided may have been responsible for the sporadic use of mobile phones for notification by some notifiers, despite the high acceptability of the strategy in general (mSIMU 2017). One study in Bangladesh engaged mothers to provide notification of births, but found the rates of maternal notification to be low. The authors suggested that this might be connected to a lack of time among mothers, who were busy taking care of new babies (mTika 2016). Finally, a study in Syria noted that it was challenging to convince partners to continue reporting on attacks on and deaths among health workers, as the availability of these data did not appear to lead to any change (MVH 2017).

###### C. Factors related to families for whom birth and death is notified via mobile devices

***Finding C.1. For some families, costs may be a barrier to completing birth and death registration post‐notification (low‐confidence finding).***

Several studies described costs incurred by families when registering and certifying births and deaths that may be prohibitive for some families. While these costs are not specific to notification conducted via mobile devices, they included transportation costs to and from the registration centre (MOVE‐IT 2013), lost wages (MBRP 2015; MBRT 2016), and penalties associated with late certification (MOVE‐IT 2013). Transportation costs in these studies included the lack of inexpensive transport options, the time required for travel, and the need for multiple trips to distant registration centres (MBRP 2015; MBRT 2016; MOVE‐IT 2013). Some studies suggested that families living farther away from registration centres or that were poorer were less likely to complete birth or death registration and certification due to cost barriers (MBRP 2015; MOVE‐IT 2013). For example, in Pakistan, the costs of birth registration were estimated to be equivalent to the average daily wage in some communities (MBRP 2015). One study pointed out that the use of mobile devices to facilitate issuance of birth certification at the site of notification would remove the need for families to travel to registration centres (MBRT 2016).

***Finding C.2. There may be a need for targeted demand generation activities in communities with low awareness of the need of birth and death registration, alongside the use of mobile phones for birth and death notification (low‐confidence finding).***

Several studies reported the need for targeted advocacy campaigns, including campaigns delivered via mobile phones, to increase awareness of the importance of timely vital registration (MBRG 2014; MBRT 2016; MOVE‐IT 2013; mVRS 2017) (although low awareness is not specific to notification conducted via mobile devices). Two studies suggested that linking birth registration with school entry requirements may lead some families to delay the process until the child is ready to begin attending school (MBRG 2014; mVRS 2017). In Tanzania, some families provided no specific reason for the failure to complete registration, even though births and deaths in these families had already been notified by the health workers using mobile phones (MOVE‐IT 2013).

***Finding C.3. Sociocultural norms may influence the timely identification of births and deaths, and should be taken into consideration when developing mobile phone interventions for notification (low‐confidence finding).***

Studies reported that sociocultural norms could create challenges for identifying births and deaths (MBRG 2014; MBRP 2015; MOVE‐IT 2013). Examples of such sociocultural norms included moving out of a district after the death of family member (MOVE‐IT 2013); delays in, or failure to record still births, abortions, or maternal deaths (MBRG 2014); preference for giving birth outside of the study area (ANISA 2016); as well as shame and stigma associated with births to unmarried women (MBRP 2015). In the ANISA study, authors also reported the unwillingness of people living in rural areas in Pakistan to be outside after sunset, limiting identification of births that occurred in the evening or night until the next morning (ANISA 2016). Again, these issues are relevant for, but not specific to, notification of birth and deaths by mobile device.

***Finding C.4. Birth and death notification may increase access to these services for some families. However, they may also increase inequities in access related to low availability of supportive infrastructure (network coverage, roads, human resources), human factors (age, gender, literacy, poverty), and selective funding priorities of donors (moderate‐confidence finding).***

A number of studies suggested that the use of mobile devices improved the reach of birth and death notification interventions to marginalised populations. For instance, a study from Kenya suggested that using community‐based lay notifiers for conducting birth notification via mobile devices facilitated the timely identification and registration of 'hard‐to‐reach' populations of rural women (Gisore 2012). Similarly, a study from Bangladesh reported successfully registering urban street children (mTika 2016). However, studies also reported inequities in the implementation of birth and death notification via mobile devices related to the availability of supportive infrastructure, human factors and availability of funding.

In Tanzania and Pakistan, where birth notification via mobile devices was conducted in areas with low birth registration rates, study authors reported prioritising regions with adequate network coverage, good roads, and access to provincial capital cities for implementation (MBRP 2015; MBRT 2016). In the MOVE‐IT project in Tanzania, some villages could not participate in the implementation of birth notification via mobile devices due to lack of network coverage or absence of a village executive officer to send notifications (MOVE‐IT 2013). A study from South Sudan also reported challenges in achieving 100% reporting of disease surveillance activities due to lack of staff and network coverage (Yugi 2016). In Rwanda, it was reported that some community health workers had to travel long distances to access facilities where they could charge their mobile devices (Ngabo 2012).

In the M‐SIMU project in Kenya, gender and education status was associated with ability to notify events using mobile phones. For example, village reporters who were female, or had higher education levels, performed better and made fewer errors compared to their male and less educated counterparts (mSIMU 2017). A study in South Sudan reported challenges in composing SMS disease surveillance notifications among health workers with lower English proficiency (Yugi 2016). Another study in Ghana reported using bilingual proctors during training sessions with health workers to accommodate Twi and English speakers (Andreatta 2011). In the mTika study in Bangladesh, where mothers were expected to send SMS notification related to the birth of their child, the authors reported that most mothers shared phones but did not own them (mTika 2016). A study in Lao PDR reported the inability of health workers in the comparison arms to afford mobile phone credit as the reason for lower rates of timely birth notifications (Xeuatvongsa 2016).

Finally, one study in Uganda reported that districts which received the greatest funding from donors also showed the greatest improvements in birth registration rates (mVRS 2017).

###### D. Factors related to government involvement in birth and death notification via mobile devices

***Finding D.1. Strong government commitment is a key factor in the successful implementation of birth and death notification via mobile devices (low‐confidence finding).***

Studies reported successful implementation of birth and death notification initiatives via mobile devices in countries where there was strong political will and support from the national government (eCRVS‐Mozambique 2017; MBRL 2011; MBRT 2016; mVRS 2017; Ngabo 2012; Yugi 2016). For instance, in Tanzania, the government’s willingness to adopt a decentralised approach to civil registration was listed as a factor driving successful implementation of the mobile birth registration initiative (MBRT 2016). Authors reported that they included government as a formal partner from the start, and solicited their input throughout the project lifecycle (MBRT 2016).

###### E. Factors related to the technologies used for birth and death notification via mobile devices

***Finding E.1. Cost is an important consideration in the purchase, set‐up, and scaling up of mobile technologies needed for birth and death notification (low‐confidence finding).***

The studies noted taking costs into consideration during the purchase, set‐up, and scaling of mobile technologies for birth and death notification (Gisore 2012; mTika 2016; mVRS 2017; Ngabo 2012; Pascoe 2012; Van Dam 2015; Xeuatvongsa 2016; Yugi 2016) The cost categories mentioned included:

- Initial costs of setting up the technology, including purchase of mobile devices for notifiers, servers, and other related technologies (MBRT 2016; mVRS 2017; Ngabo 2012; Pascoe 2012; Xeuatvongsa 2016);
- Purchasing airtime for notifiers or paying for costs of information transmission (e.g. costs of SMS) (Ngabo 2012; Pascoe 2012; Xeuatvongsa 2016).

Some studies reported that the operating costs of using mobile phones reduced as the project progressed beyond the initial investment and technology set‐up phase, although reasons for this reduction were not given (Gisore 2012; MOVE‐IT 2013; mVRS 2017).

Some strategies to reduce technology costs discussed in the studies included:

- Using notifiers’ own phones (mTika 2016; Xeuatvongsa 2016);
- Public‐private partnerships with mobile network operators for cheaper rates on SMS or data‐related expenses (Ngabo 2012);
- Planning for communication costs in monthly project or health worker budgets (Yugi 2016).

***Finding E.2. Challenges when notifying births and deaths via mobile devices include poor access to electricity and incompatibility with existing systems (low‐confidence finding).***

The studies reported several challenges related to using and maintaining mobile devices, which in turn impacted the ability of health workers to provide birth and death notifications. Challenges described included:

- Access to electricity for charging devices (Ngabo 2012; Pascoe 2012);
- Reliable electricity to maintain servers, in part due to frequent power cuts (MBRL 2011; MBRP 2015);
- Insufficient phone memory for storing data (MBRT 2016; Pascoe 2012);
- Accidentally erasing apps related to birth or death notification (Pascoe 2012);
- Lost devices (Gisore 2012; Pascoe 2012);
- New developments in technology and the need for newer technology (MBRT 2016; Pascoe 2012);
- Incompatibility with existing systems (MBRT 2016).

***Finding E.3. The availability of network connectivity is a key factor in the successful implementation and scale‐up of birth and death notification via mobile devices (moderate‐confidence finding).***

Most studies reported the high coverage of mobile networks in the study areas as the key reason for implementing notification of vital events via mobile devices (ANISA 2016; MBRT 2016; mSIMU 2017; mVRS 2017; Ngabo 2012). However, other studies reported challenges in implementing or scaling mobile phone‐based notification of vital events in areas with poor network coverage (Pascoe 2012; Xeuatvongsa 2016; Yugi 2016). Two studies in Tanzania reported overcoming barriers related to poor or unreliable network coverage by using a system that was capable of offline data collection and storage. This allowed data to be transmitted when a network connection was available (MBRT 2016; Pascoe 2012). Upon implementing a system capable of offline data collection, Pascoe and colleagues reported that messages would queue for submission when offline, and health workers had to find locations with good mobile network coverage to upload the data (Pascoe 2012).

***Finding E.4: Data security and encryption measures are needed to preserve the confidentiality of birth and death information notified via mobile devices (low‐confidence finding).***

The included studies described a variety of data security measures including:

- Use of dedicated usernames and passwords (MVH 2017; Ngabo 2012; Van Dam 2015);
- Anonymous reporting of sensitive data (MVH 2017);
- Data encryption and secure protocols for data transmission (MVH 2017; Van Dam 2015);
- Limiting user permissions to view or edit data (MBRT 2016; MVH 2017; Ngabo 2012); and
- Ability to lock and wipe phone remotely if phone is lost (MBRT 2016).

In addition, one study from Zambia described the ability to produce de‐identified reports from data, which could be subsequently used for research (Van Dam 2015).

## Discussion

### Summary of main results

We identified only one study, focusing on birth notification, which met the eligibility criteria for inclusion in relation to the review's primary objective. Based on this study, we are uncertain about the impacts of birth notification via mobile devices as the certainty of the evidence is very low. No studies evaluating the effectiveness of death notification via mobile devices were eligible for inclusion in the review.

In relation to the review's secondary objectives, we identified a number of studies that helped us summarise factors that could influence the implementation of birth and death notification via mobile devices. These studies showed that a wide range of factors appear to influence the implementation of this approach, including issues tied to the health system and the notification system, the person responsible for notifying, the community, and the families involved.

All 21 studies of birth or death notification via mobile phones were from low‐ or middle‐income countries. This can probably be explained by the use of more robust health information and civil registration and vital statistics systems in high‐income countries, making it unnecessary to use mobile phones for birth and death notification.

#### Description of interventions

We found significant heterogeneity in the implementation of birth and death notification via mobile devices. Most studies used lay health workers, community informants, healthcare organisations, or families/individuals as notifiers of birth and death. The emphasis, in many cases, was on the use of simple technologies (e.g. basic phones, SMS or voice‐based notification), although we found examples of smartphone app‐based data collection for birth and death notification.

### Overall completeness and applicability of evidence

This review is a comprehensive assessment of the evidence published since 2000 on birth and death notification via mobile devices. The findings of this review suggest that there is a paucity of well conducted studies on the effectiveness of birth and death notification via mobile devices (primary review objective). We used a multi‐pronged search strategy including peer‐reviewed and grey literature, solicitation of relevant studies from the digital health community, and a review of trial registration databases to improve the chances of identifying published, unpublished and ongoing studies. We therefore anticipate that we identified all eligible studies of the effectiveness of birth and death notification via mobile devices. Because the evidence that we identified was limited in nature and of very low certainty, we cannot meaningfully discuss the applicability of this evidence. Outcome measures like coverage (e.g. proportion) of births or deaths notified via mobile devices may not be indicators of intervention impact if overall coverage of births or deaths notification does not increase. In future iterations of the review, authors may wish to consider additional outcome measures such as the vital statistics performance index.

#### Certainty of the evidence

Based on the GRADE approach, the certainty of evidence for the birth notification outcomes was very low, and this was related to the non‐randomised study design used and concerns regarding precision and directness. As noted above, we did not identify any eligible studies evaluating the effectiveness of death notification via mobile devices.

For the findings based on the studies included in the review of the secondary objectives, confidence in the evidence, based on the GRADE‐CERQual approach, was low to moderate. The main reasons for downgrading related to the methodological limitations of the studies and concerns regarding adequacy of the evidence.

### Potential biases in the review process

For the primary objectives, we attempted to minimise potential biases in the review process by adhering to Cochrane (Higgins 2011) and EPOC guidance (EPOC 2017a). We conducted comprehensive searches without limiting the searches to a specific language, and two review authors independently assessed study eligibility, extracted data, and assessed the risk of bias for each included study.

For the secondary review objective, we followed Cochrane and EPOC guidance for qualitative evidence syntheses (Glenton 2019, Noyes 2018) and also undertook comprehensive searches and used two review authors to independently assess study eligibility, extract data, and assess the methodological limitations of each included source. However, as more of these studies may be available through the grey literature only, or may be unpublished, it is possible that some were missed in our search process. Also, we used a newly developed tool (the WEIRD tool) to assess the limitations of some of the sources, and it is not yet completely clear how best to apply this tool (Lewin 2019).

### Agreements and disagreements with other studies or reviews

There is one previous review of birth and death notification (WHO 2013a). The main differences between this review and the earlier review are that our review:

- Limited the intervention to those delivered via mobile devices only. The previous review included any e‐health intervention.
- Expanded the definition of the intervention to use cases outside the realm of national civil registration and vital statistics systems. The previous review focussed on civil registration and vital statistics systems exclusively.
- Used a more comprehensive search strategy inclusive of the grey literature.
- Used the EPOC review group methods and approaches to reduce bias in development and implementation of the review protocol.

Despite these differences, our finding that there are very few rigorous studies of the effectiveness of birth and death notification via mobile devices agrees with that of the earlier review. In addition, a qualitative evidence synthesis of healthcare workers’ perceptions and experience of using mHealth technologies for delivering primary healthcare services has now been published (Odendaal 2020). The findings of this synthesis complement those of this review and contributed to the Implications for practice section.

## Authors' conclusions

**Implications for practice**

Below are a set of questions that may help health system or programme managers when implementing or planning for birth or death notification via mobile devices. These questions are also summarised in Appendix 6. These questions build on the findings of this review, and are also partly based on similar implications for practice from a linked review on health workers’ perceptions and experiences of using mHealth technologies to deliver primary healthcare services (Odendaal 2020).

**1. Have you taken the needs and view of notifiers and community members into account when developing and planning the birth or death notification system?**

- Will you involve the person responsible for notifying births and deaths via mobile device (the ‘notifier’) in the planning, development and evaluation of the mobile application specifically and the birth‐death notification process in general?
- Is there a need to raise awareness in your community about the importance of timely birth and death registration?
- Have you taken people’s views and customs regarding birth and death into account when developing routines for birth‐death notification? For instance, are you likely to find relevant family members at home and accessible after a birth or a death? Are people likely to volunteer information about certain types of births and deaths, such as stillbirths, babies born to unmarried mothers, or suicides?

**2. Do notifiers have the legal authority to provide the services expected of them?**

- For instance, are they legally able to issue birth/death certificates as well as notifications? Will they have access to relevant databases? Will you need to work with the government to make any necessary changes to the law? Or should you reconsider your choice of notifier?

**3. Will the planned notifiers have enough time within their current roles to deliver birth‐death notification services timeously?**

- For instance, where health facility staff are notifiers, do they consider birth‐death notification to be part of their job? If this task is to be added to existing tasks, will they be sufficiently compensated, for instance if their job is now extended to include home visits?

**4. Will families have reasonable access to notifiers and to post‐notification services?**

- Will families and notifiers be able to reach each other easily so that the necessary information can be collected in a timely way? For instance, where the notifier is expected to travel to families, does he or she have access to reliable transport? Where families are expected to travel to the notifier, is this a reasonable distance and do they have access to reliable and affordable transport?
- Will families be able to access post‐notification services, for instance birth or death certifications or childhood vaccinations, in an easy and timely manner? Have you considered increasing the number or proximity of service points where registration can take place? Have you considered whether birth certificates can be issued at the time and place of notification to reduce the need for parents to travel to a registration centre?

**5. Are there systems in place to analyse birth and death data to identify important health problems and trends?**

- Are there systems in place to regularly analyse incoming data around births and deaths so that you can quickly identify important problems, trends or changes in people’s health? Where important issues are identified, is there a plan for how these will be addressed?

**6. Have the costs to the health system and to notifiers been included in the budget?**

- When budgeting for birth‐death notification systems using mobile devices, have you considered the initial costs of setting up the technology (including purchase of mobile devices for notifiers, servers, and other related technologies) as well as running costs (including purchasing airtime for notifiers or paying for costs of information transmission)?
- If notifiers are expected to use their own mobile phones, how will their costs be covered? For instance, will they be provided with phone credit, and how will you ensure that this phone credit is sufficient and timely?

**7. Have you assessed and taken into account the technological requirements for notifiers and for existing electronic health information systems?**

- Will notifiers have easy and reliable access to networks and to electricity to charge devices? Where offline data coverage and storage is used in settings with poor network coverage, is it easy for notifiers to find locations with good network coverage to upload data at a later date?
- Will notifiers have access to reliable mobile devices with sufficient memory for storing data? Are these devices easy to repair or replace locally, and who will pay for this?
- Will your birth‐death notification system be linked to or integrated into other relevant systems, such as existing electronic health information systems, and does it have clear government support? And have you considered the requirements to ensure interoperability?
- How will you ensure the confidentiality of the birth and death information? Have you considered security measures for any mobile devices used to collect and transmit data, mechanisms of data encryption at rest and transmission, and access to secure data servers? For instance, have you considered the use of dedicated usernames and passwords, anonymous reporting of sensitive data, data encryption and secure protocols of data transmission, using access control to limit user permissions to view or edit data, ability to lock and wipe mobile phone remotely if lost, and data storage in a tier 1 high security data centre?

**8. Will the planned birth or death notification system reduce rather than increase inequities?**

- Are there groups of people in your community who may find it difficult to benefit from a birth‐death notification system via mobile device, for instance, because of poor network coverage, poor roads, lack of staff, language or literacy issues, or long distances to post‐notification services? If so, what strategies will you use to ensure that these groups do not fall behind?

**9. Is there a plan for addressing the training needs of notifiers?**

- Do you know enough about notifiers’ training needs? Notifiers may be very familiar with paper‐based systems for notification, but how familiar are they with the use of mobile phones? What kind of language skills and literacy rates are they likely to have?
- Will you have regular training opportunities for notifiers, including notifiers coming in to the programme for training refreshers at different time points?
- What kind of follow‐up support will you be offering notifiers? For instance, where can notifiers go when they have questions or problems about the technology used or the process of notification?
- Will you have access to people locally that have the skills, the mandate and the availability to offer training and support?

**10. Is there a plan for monitoring notifiers and providing supportive supervision?**

- How do you plan to encourage and support notifiers to ensure that they submit high‐quality timely data? For instance, will notifiers receive acknowledgements that their data has been received? Will they receive reminders? Do you plan to have regular performance meetings? Where the data that they and others have collected have allowed you to identify problems and develop strategies, will notifiers be made aware of their own contribution?
- How do you plan to monitor the quality and timeliness of the data collected by notifiers? For instance, will you check all data continuously or only carry out spot checks?

**Implications for research**

We need well conducted evaluations of the effectiveness of birth notification and death notification via mobile devices. Given the challenges of setting up randomised trials of these health‐system level interventions, researchers should consider other study designs that include some form of comparison group or a sequence of observations over time. These could include controlled before‐after studies (with at least two intervention sites and two control sites) or interrupted time series studies (with clearly defined time points when the intervention occurred and at least three data points before and three after the intervention). If implemented in the context of health systems, these effectiveness studies should clearly indicate the contribution of (timely) birth notification to coverage and timeliness of follow‐on health services such as immunisations. Studies of the effects of death notification should also look at the impact of the notification on activities such as disease surveillance and verbal autopsy. Furthermore, these effectiveness studies should collect data on the costs related to purchasing, set‐up and scaling of mobile devices and supporting technologies. We also need more research on factors that may affect the implementation of birth‐death notification via mobile devices. Researchers should consider using qualitative study designs to explore this question. They should also consider exploring programmes that have been implemented at scale and that have been running for some time, rather than focussing only on start‐up or small‐scale programmes. Our review identified a number of factors that may influence the implementation of birth‐death notification via mobile device. However, our confidence in several of these findings is low, often due to the methodological limitations of the underlying research. Factors that may need further exploration include:

- Modifications to legal frameworks governing civil registration so as to allow notification via mobile device and the inclusion of new cadres of notifiers.
- Ways of integrating birth and death notification via mobile devices with underlying health and civil registration systems.
- Different approaches to strengthening capacity to train local notifiers, and to expanding the range of cadres who can conduct birth and death notification.
- How birth and death notification via mobile devices can be used to facilitate provider and health system accountability for the collection of vital data and for post‐notification service delivery.
- Strategies for maintaining and updating the mobile devices needed to notify births and deaths.
- Strategies for mitigating costs that may act as barriers to families using post‐notification services.
- Ways of advocating in communities regarding the need for timely birth and death registration, including via mobile devices, and that take into account local sociocultural norms and concerns about the confidentiality of information.
- Strategies for ensuring that the implementation of these interventions reduces inequities through reaching under‐registered populations.

## History

Protocol first published: Issue 1, 2018

## Appendices

### Appendix 1. Search strategies

**MEDLINE and Epub Ahead of Print, In‐Process & Other Non‐Indexed Citations and Daily 1946 to August 01, 2019, Ovid**

**Table:**

**#**	**Searches**	**Results**
1	Cell Phones/	7897
2	Smartphone/	3142
3	MP3‐Player/	178
4	Computers, Handheld/	3382
5	((cell* or mobile*) adj1 (phone* or telephone* or technolog* or device*)).ti,ab,kw.	17088
6	(handheld or hand‐held).ti,ab,kw.	11646
7	(smartphone* or smart‐phone* or cellphone* or mobiles).ti,ab,kw.	10157
8	((personal adj1 digital) or (PDA adj3 (device* or assistant*)) or MP3 player* or MP4 player*).ti,ab,kw.	1340
9	(samsung or nokia).ti,ab,kw.	1077
10	(windows adj3 (mobile* or phone*)).ti,ab,kw.	50
11	android.ti,ab,kw.	2202
12	(ipad* or i‐pad* or ipod* or i‐pod* or iphone* or i‐phone*).ti,ab,kw.	2570
13	(tablet* adj3 (device* or computer*)).ti,ab,kw.	1422
14	Telemedicine/	19941
15	Videoconferencing/ or Webcasts as topic/	1664
16	Text Messaging/	2331
17	Telenursing/	200
18	(mhealth or m‐health or "mobile health" or ehealth or e‐health or "electronic health").ti,ab,kw.	23204
19	(telemedicine or tele‐medicine or telehealth or tele‐health or telecare or tele‐care or telenursing or tele‐nursing or telepsychiatry or tele‐psychiatry or telemonitor* or tele‐monitor* or teleconsult* or tele‐consult* or telecounsel* or tele‐counsel* or telecoach* or tele‐coach*).ti,ab,kw.	16709
20	(videoconferenc* or video‐conferenc* or webcast* or web‐cast*).ti,ab,kw.	2828
21	((text* or short or voice or multimedia or multi‐media or electronic) adj1 messag*).ti,ab,kw.	4501
22	(texting or texted or texter* or ((sms or mms) adj (service* or messag*)) or interactive voice response* or IVR or voice call* or callback*).ti,ab,kw.	3068
23	(Facebook or Twitter or Whatsapp* or Skyp* or YouTube or "You Tube").ti,ab,kw.	6738
24	Mobile Applications/	4365
25	"mobile app*".ti,ab,kw.	3678
26	Social Media/	6166
27	(social adj (media or network*)).ti,ab,kw.	22778
28	Reminder Systems/	3247
29	(remind* adj3 (text* or system* or messag*)).ti,ab,kw.	1672
30	Electronic Mail/	2559
31	(electronic mail* or email* or e‐mail or webmail).ti,ab,kw.	14143
32	Medical informatics/ or Medical informatics applications/	13486
33	Nursing informatics/ or Public health informatics/	2601
34	((medical or clinical or health or healthcare or nurs*) adj3 informatics).ti,ab,kw.	5318
35	Multimedia/	1855
36	Hypermedia/	396
37	Blogging/	930
38	(multimedia or multi‐media or hypermedia or hyper‐media or blog* or vlog* or weblog* or web‐log*).ti,ab,kw.	7023
39	Interactive Tutorial/	265
40	Computer‐Assisted Instruction/	11537
41	((interactive or computer‐assisted) adj1 (tutor* or technolog* or learn* or instruct* or software or communication)).ti,ab,kw.	2421
42	or/1‐41	167770
43	registries/ or hospital records/ or electronic health records/ or vital statistics/	106031
44	data collection/ or records as topic/ or birth certificates/ or death certificates/ or medical records/ or medical record linkage/ or medical records systems, computerized/ or vital statistics/ or mortality/ or child mortality/ or fetal mortality/ or infant mortality/ or perinatal mortality/ or maternal mortality/ or mortality, premature/ or information management/ or health information management/ or health information exchange/ or "information storage and retrieval"/	282375
45	(birth adj3 (registr* or notif* or report* or record* or log* or certif* or collection or survey* or surveillance)).ti,ab,kw.	10816
46	(((death* or mortality or vital) adj3 (registr* or notif* or report* or record* or log* or certif* or collection or survey* or surveillance)) or verbal autops*).ti,ab,kw.	47010
47	or/43‐46	415123
48	randomized controlled trial.pt.	486501
49	random*.tw.	1065256
50	intervention*.tw.	907477
51	control*.tw.	3621320
52	evaluat*.tw.	3268041
53	effect*.tw.	6497227
54	impact.tw.	872233
55	(time series or time point?).tw.	133906
56	repeated measur*.tw.	42428
57	or/48‐56	11297873
58	case reports.pt.	2035589
59	Case‐Control Studies/	268199
60	(case study or case studies or case control stud* or case report?).tw.	521554
61	or/58‐60	2480427
62	57 or 61	13148845
63	exp Animals/	22493520
64	Humans/	17888818
65	63 not (63 and 64)	4604702
66	review.pt.	2541529
67	meta analysis.pt.	103401
68	news.pt.	196305
69	comment.pt.	790990
70	editorial.pt.	498709
71	cochrane database of systematic reviews.jn.	14292
72	comment on.cm.	790935
73	(systematic review or literature review).ti.	137282
74	or/65‐73	8322433
75	62 not 74	9532121
76	42 and 47 and 75	8340

**Embase 1974 to 2019 Week 30, Ovid**

**Table:**

**#**	**Searches**	**Results**
1	mobile phone/ or smartphone/	24128
2	mp3 player/	190
3	((cell* or mobile*) adj1 (phone* or telephone* or technolog* or device*)).ti,ab,kw.	21938
4	(handheld or hand‐held).ti,ab,kw.	16007
5	(smartphone* or smart‐phone* or cellphone* or mobiles).ti,ab,kw.	14066
6	((personal adj1 digital) or (PDA adj3 (device* or assistant*)) or MP3 player* or MP4 player*).ti,ab,kw.	1834
7	(samsung or nokia).ti,ab,kw.	1967
8	(windows adj3 (mobile* or phone*)).ti,ab,kw.	75
9	android.ti,ab,kw.	3473
10	(ipad* or i‐pad* or ipod* or i‐pod* or iphone* or i‐phone*).ti,ab,kw.	4771
11	(tablet* adj3 (device* or computer*)).ti,ab,kw.	2261
12	telemedicine/ or telecardiology/ or teleconsultation/ or teledermatology/ or telediagnosis/ or telemonitoring/ or telepathology/ or telepsychiatry/ or teleradiotherapy/ or telesurgery/ or teletherapy/	33916
13	videoconferencing/ or webcast/	3698
14	text messaging/	4217
15	telenursing/	250
16	(mhealth or m‐health or "mobile health" or ehealth or e‐health or "electronic health").ti,ab,kw.	30985
17	(telemedicine or tele‐medicine or telehealth or tele‐health or telecare or tele‐care or telenursing or tele‐nursing or telepsychiatry or tele‐psychiatry or telemonitor* or tele‐monitor* or teleconsult* or tele‐consult* or telecounsel* or tele‐counsel* or telecoach* or tele‐coach*).ti,ab,kw.	22713
18	(videoconferenc* or video‐conferenc* or webcast* or web‐cast*).ti,ab,kw.	4181
19	(((text* or short or voice or multimedia or multi‐media or electronic or instant) adj1 messag*) or instant messenger).ti,ab,kw.	6337
20	(texting or texted or texter* or ((sms or mms) adj (service* or messag*)) or interactive voice response* or IVR or voice call* or callback* or voice over internet or VOIP).ti,ab,kw.	4531
21	(Facebook or Twitter or Whatsapp* or Skyp* or YouTube or "You Tube" or Google Hangout*).ti,ab,kw.	9794
22	mobile application/	8560
23	"mobile app*".ti,ab,kw.	4381
24	social media/	16070
25	(social adj (media or network*)).ti,ab,kw.	30222
26	reminder system/	2459
27	(remind* adj3 (text* or system* or messag*)).ti,ab,kw.	2439
28	e‐mail/	19364
29	(electronic mail* or email* or e‐mail or webmail).ti,ab,kw.	28776
30	medical informatics/	19647
31	nursing informatics/	1489
32	((medical or clinical or health or healthcare or nurs*) adj3 informatics).ti,ab,kw.	8429
33	multimedia/	3712
34	hypermedia/	379
35	blogging/	290
36	(multimedia or multi‐media or hypermedia or hyper‐media or blog* or vlog* or weblog* or web‐log*).ti,ab,kw.	10549
37	teaching/	85923
38	((interactive or computer‐assisted) adj1 (tutor* or technolog* or learn* or instruct* or software or communication)).ti,ab,kw.	3515
39	or/1‐38	315569
40	birth certificate/	2373
41	death certificate/	8186
42	registration/ or register/	139680
43	vital statistics/	3437
44	information processing/	228367
45	electronic data interchange/ or electronic medical record system/ or information storage/	3471
46	information system/ or clinical data repository/ or hospital information system/ or medical information system/ or nursing information system/	75329
47	medical record/ or electronic patient record/	167774
48	(birth adj3 (registr* or notif* or report* or record* or log* or certif* or collection or survey* or surveillance)).ti,ab,kw.	13790
49	(((death* or mortality or vital) adj3 (registr* or notif* or report* or record* or log* or certif* or collection or survey* or surveillance)) or verbal autops*).ti,ab,kw.	67583
50	or/40‐49	651261
51	Randomized Controlled Trial/	561329
52	Controlled Clinical Trial/	464222
53	Quasi Experimental Study/	5856
54	Pretest Posttest Control Group Design/	401
55	Time Series Analysis/	23631
56	Experimental Design/	17260
57	Multicenter Study/	223169
58	(randomis* or randomiz* or randomly).ti,ab.	1178255
59	groups.ab.	2682873
60	(trial or multicentre or multicenter or multi centre or multi center).ti.	335596
61	(intervention? or controlled or control group? or (before adj5 after) or (pre adj5 post) or ((pretest or pre test) and (posttest or post test)) or quasiexperiment* or quasi experiment* or evaluat* or effect? or impact? or time series or time point? or repeated measur*).ti,ab.	11573509
62	(case study or case studies or case report?).mp.	2565231
63	or/51‐62	15033571
64	exp animals/ or exp invertebrate/ or animal experiment/ or animal model/ or animal tissue/ or animal cell/ or nonhuman/	26220545
65	human/ or normal human/ or human cell/	20018599
66	64 and 65	19961247
67	64 not 66	6259298
68	(systematic review or literature review).ti.	164383
69	"cochrane database of systematic reviews".jn.	13466
70	or/67‐69	6435633
71	63 not 70	12001313
72	39 and 50 and 71	15815
73	limit 72 to embase	5609

**CENTRAL, Cochrane Library**

**Table:**

ID	Search	Hits
#1	MeSH descriptor: [Cell Phone] this term only	620
#2	MeSH descriptor: [Smartphone] this term only	250
#3	MeSH descriptor: [MP3‐Player] this term only	21
#4	MeSH descriptor: [Computers, Handheld] this term only	239
#5	((cell* or mobile*) near/1 (phone* or telephone* or technolog* or device*)):ti,ab,kw	3495
#6	(handheld or hand‐held):ti,ab,kw	1984
#7	(smartphone* or smart‐phone* or cellphone* or mobiles):ti,ab,kw	2603
#8	((personal near/1 digital) or (PDA near/3 (device* or assistant*)) or MP3 player* or MP4 player*):ti,ab,kw	286
#9	(samsung or nokia):ti,ab,kw	115
#10	(windows near/3 (mobile* or phone*)):ti,ab,kw	4
#11	android:ti,ab,kw	478
#12	(ipad* or i‐pad* or ipod* or i‐pod* or iphone* or i‐phone*):ti,ab,kw	771
#13	(tablet* near/3 (device* or computer*)):ti,ab,kw	609
#14	MeSH descriptor: [Telemedicine] this term only	1741
#15	MeSH descriptor: [Videoconferencing] this term only	160
#16	MeSH descriptor: [Webcasts as Topic] this term only	21
#17	MeSH descriptor: [Text Messaging] this term only	664
#18	MeSH descriptor: [Telenursing] this term only	28
#19	(mhealth or m‐health or "mobile health" or ehealth or e‐health or "electronic health"):ti,ab,kw	3598
#20	(telemedicine or tele‐medicine or telehealth or tele‐health or telecare or tele‐care or telenursing or tele‐nursing or telepsychiatry or tele‐psychiatry or telemonitor* or tele‐monitor* or teleconsult* or tele‐consult* or telecounsel* or tele‐counsel* or telecoach* or tele‐coach*):ti,ab,kw	5018
#21	(videoconferenc* or video‐conferenc* or webcast* or web‐cast*):ti,ab,kw	664
#22	(((text* or short or voice or multimedia or multi‐media or electronic or instant) near/1 messag*) or instant messenger) .ti,ab,kw	53
#23	(texting or texted or texter* or ((sms or mms) near (service* or messag*)) or interactive voice response* or IVR or voice call* or callback* or voice over internet or VOIP):ti,ab,kw	2361
#24	(Facebook or Twitter or Whatsapp* or Skyp* or YouTube or "You Tube" or Google Hangout*):ti,ab,kw	762
#25	MeSH descriptor: [Mobile Applications] this term only	420
#26	"mobile app*":ti,ab,kw	393
#27	MeSH descriptor: [Social Media] this term only	108
#28	(social near (media or network*)):ti,ab,kw	2162
#29	MeSH descriptor: [Reminder Systems] this term only	857
#30	(remind* near/3 (text* or system* or messag*)):ti,ab,kw	1824
#31	MeSH descriptor: [Electronic Mail] this term only	304
#32	(electronic mail* or email* or e‐mail or webmail):ti,ab,kw	4062
#33	MeSH descriptor: [Medical Informatics] this term only	72
#34	MeSH descriptor: [Medical Informatics Applications] this term only	23
#35	MeSH descriptor: [Nursing Informatics] this term only	10
#36	MeSH descriptor: [Public Health Informatics] this term only	1
#37	((medical or clinical or health or healthcare or nurs*) near/3 informatics):ti,ab,kw	311
#38	MeSH descriptor: [Multimedia] this term only	212
#39	MeSH descriptor: [Hypermedia] this term only	8
#40	MeSH descriptor: [Blogging] this term only	13
#41	(multimedia or multi‐media or hypermedia or hyper‐media or blog* or vlog* or weblog* or web‐log*):ti,ab,kw	1227
#42	MeSH descriptor: [Interactive Tutorial] this term only	0
#43	MeSH descriptor: [Computer‐Assisted Instruction] this term only	1179
#44	((interactive or computer‐assisted) near/1 (tutor* or technolog* or learn* or instruct* or software or communication)):ti,ab,kw	1442
#45	{or #1‐#44}	26519
#46	MeSH descriptor: [Registries] this term only	881
#47	MeSH descriptor: [Hospital Records] this term only	15
#48	MeSH descriptor: [Electronic Health Records] this term only	309
#49	MeSH descriptor: [Vital Statistics] this term only	3
#50	MeSH descriptor: [Data Collection] this term only	1218
#51	MeSH descriptor: [Records] this term only	34
#52	MeSH descriptor: [Birth Certificates] this term only	4
#53	MeSH descriptor: [Death Certificates] this term only	11
#54	MeSH descriptor: [Medical Records] this term only	727
#55	MeSH descriptor: [Medical Record Linkage] this term only	29
#56	MeSH descriptor: [Medical Records Systems, Computerized] this term only	197
#57	MeSH descriptor: [Mortality] this term only	483
#58	MeSH descriptor: [Child Mortality] this term only	59
#59	MeSH descriptor: [Fetal Mortality] this term only	2
#60	MeSH descriptor: [Infant Mortality] this term only	557
#61	MeSH descriptor: [Perinatal Mortality] this term only	87
#62	MeSH descriptor: [Maternal Mortality] this term only	107
#63	MeSH descriptor: [Mortality, Premature] this term only	3
#64	MeSH descriptor: [Information Management] this term only	16
#65	MeSH descriptor: [Health Information Management] this term only	6
#66	MeSH descriptor: [Health Information Exchange] this term only	5
#67	MeSH descriptor: [Information Storage and Retrieval] this term only	115
#68	(birth near/3 (registr* or notif* or report* or record* or log* or certif* or collection or survey* or surveillance)):ti,ab,kw	707
#69	(((death* or mortality or vital) near/3 (registr* or notif* or report* or record* or log* or certif* or collection or survey* or surveillance)) or verbal autops*):ti,ab,kw	4993
#70	{or #46‐#69}	10116
#71	#45 and #70	823

**POPLINE, K4Health**

Keyword:

TEXT MESSAGING OR MOBILE DEVICES OR INFORMATION COMMUNICATION TECHNOLOGY OR CELLULAR PHONE

**OR**

All Fields:

((cell OR cellular OR mobile) AND (phone OR phones OR telephone OR telephones OR technology OR technologies OR device OR devices)) OR smartphone OR smartphones OR smart‐phone OR smart‐phones OR cellphone OR cellphones OR mobiles OR mhealth OR m‐health OR "mobile health" OR ehealth OR e‐health OR "electronic health" OR telemedicine OR tele‐medicine OR telehealth OR tele‐health OR telecare OR tele‐care OR telenursing OR tele‐nursing OR telepsychiatry OR tele‐psychiatry OR telemonitor OR telemonitoring OR tele‐monitor OR tele‐monitoring OR teleconsult OR teleconsulting OR tele‐consult OR tele‐consulting OR telecounsel OR telecounseling OR tele‐counsel OR tele‐counseling OR telecoach OR telecoaching OR tele‐coach OR tele‐coaching OR videoconference OR videoconferences OR videoconferencing OR video‐conference OR video‐conferences OR video‐conferencing OR webcast OR webcasts OR webcasting OR web‐cast OR web‐casts OR web‐casting OR ((text OR texts OR texting OR short OR voice OR multimedia OR multi‐media OR electronic OR instant) AND (message OR messages OR messaging)) OR "instant messenger" OR texting OR texted OR texter OR texters OR ((sms OR mms) AND (service OR services OR message OR messages OR messaging)) OR "interactive voice response" OR "interactive voice responses" OR ivr OR "voice call" OR "voice calls" OR callback OR "voice over internet" OR voip OR "mobile app" OR "mobile apps" OR "mobile application" OR "mobile applications" OR "social media" OR ((medical OR clinical OR health OR healthcare OR nurse OR nurses OR nursing) AND informatics)

**AND**

All Fields:

birth* AND (registr* OR notif* OR report* OR record* OR log* OR certif* OR collection OR survey* OR surveillance)

**OR**

All Fields:

(((death* OR mortality OR vital) AND (registr* OR notif* OR report* OR record* OR log* OR certif* OR collection OR survey* OR surveillance)) OR "verbal autops*")

**OR**

Keyword:

BIRTH RECORDS OR DEATH RECORDS OR VITAL STATISTICS OR NOTIFICATION

**Web of Science Core Collection 1987‐ present, Clarivate Analytics**

Ahmad 2016, Andreatta 2011, Bose 2016, Durrani 2019, Elamein 2017, Gibson 2017, Gisore 2012. Islam 2016, Moshabela 2015, Uddin 2016, van Dam 2016, Xeautvongsa 2016

**Global Index Medicus/Global Health Library, WHO**

(mh:(("cell phones" OR smartphone OR mp3‐player OR "Computers, Handheld" OR telemedicine OR Videoconferencing OR "Text Messaging" OR Telenursing OR "Mobile Applications" OR "Reminder Systems" OR "Electronic Mail" OR "Medical Informatics" OR "Nursing Informatics" OR "Public Health Informatics" OR Multimedia OR Hypermedia OR Blogging OR Telemedicine))) OR (tw:(("cell phone" OR "cell phones" OR "cellular phone" OR "cellular phones" OR "mobile phone" OR "mobile phones" OR "mobile devices" OR "mobile devices" OR smartphone OR smartphones OR smart‐phone OR smart‐phones OR cellphone OR cellphones))) AND ((birth* AND (registr* OR notif* OR report* OR record* OR log* OR certif* OR collection OR survey* OR surveillance)) OR (((death* OR mortality OR vital) AND (registr* OR notif* OR report* OR record* OR log* OR certif* OR collection OR survey* OR surveillance)) OR "verbal autops*") OR (mh: "Birth Certificates" OR "Death Certificates"))

**International Clinical Trials Registry Platform (ICTRP), WHO**

Three separate strategies. Used advanced search, with recruitment status: All

**Strategy 1:**

Title: birth OR death OR mortality

AND

Intervention: mobile device OR mobiles OR smartphone OR phone OR cellphone

**Strategy 2:**

Title: mobile device OR mobiles OR smartphone OR phone OR cellphone

AND

Condition: birth OR death OR mortality

**Strategy 3:**

Title: registry OR registration OR records OR report OR reporting OR certificate OR certification OR log OR logs OR notification OR vital statistics

AND

Condition: birth OR death OR mortality

AND

Intervention: mobile device OR mobiles OR smartphone OR phone OR cellphone

**ClinicalTrials.gov, NIH**

Other Terms: (birth OR births OR death OR deaths OR mortality) AND ("mobile phone" OR "mobile phones" OR "mobile devices" OR mobiles OR smartphone OR smartphones)

**Epistemonikos**

Used advanced search with three separate strategies

**Strategy 1: Notification AND birth**

(title:(Birth AND notification) OR abstract:(Birth AND notification))

**Strategy 2: Notification AND death**

(title:(death AND notification) OR abstract:(death AND notification))

**Strategy 3: Birth registration**

(title:(birth registration) OR abstract:(birth registration))

**mHealthEvidence.org**

Contributed content curated by database administrator and list of records provided to review authors.

### Appendix 2. Key domains of the SURE framework

**Table:**

**Level**	**Factors affecting implementation**
*Recipients of care*	Knowledge and skills
	Attitudes regarding programme acceptability, appropriateness and credibility
	Motivation to change or adopt new behavior
*Providers of care*	Knowledge and skills
	Attitudes regarding programme acceptability, appropriateness and credibility
	Motivation to change or adopt new behavior
*Other stakeholders* *(including other healthcare providers, community health committees, community leaders, programme managers, donors, policymakers and opinion leaders)*	Knowledge and skills
	Attitudes regarding programme acceptability, appropriateness and credibility
	Motivation to change or adopt new behavior
*Health system constraints*	Accessibility of care
	Financial resources
	Human resources
	Educational and training system, including recruitment and selection
	Clinical supervision, support structures and guidelines
	Internal communication
	External communication
	Allocation of authority
	Accountability
	Community participation
	Management and/or leadership
	Information systems
	Scale of private sector care
	Facilities
	Patient flow processes
	Procurement and distribution systems
	Incentives
	Bureaucracy
	Relationships with norms and standards
*Social and political constraints*	Ideology
	Governance
	Short‐term thinking
	Contracts
	Legislation or regulation
	Donor policies
	Influential people
	Corruption
	Political stability and commitment

### Appendix 3. WEIRD criteria

**Table:**

**Item number**	**Assessment criteria^1^**	**Apply to source type**	**Questions to consider**
	**Pre‐assessment question:** Is the source material based on, or does it include, empirical data (i.e. information collected through measurement or observation)?	All source materials	None
	**Pre‐assessment question:** Please select the type of source material to which the assessment tool will be applied. Choose from the following: Description of a programme or intervention or policy or reform (e.g. a health or welfare or environmental programme or intervention) Description of the implementation of a programme or intervention or policy or reform Description of a policy process or an aspect of this process Commentary on a programme or intervention or policy or reform (e.g. a health systems or development sector policy or reform) Other [please describe]:	All source materials	None
1.	**Is there a clearly stated aim, objective or purpose for the source material?**	All source materials	• Does the source material state its aim, objective or purpose clearly? • If the aim, objective or purpose is not stated clearly by the authors, can it be derived from the material?
2.	**Is there a clear description of the source of the information reported (transparency)?**	All source materials	• Are the sources (key informants, own experience, research study etc.) described? • Where applicable, is there a clear description of who collected the information?
3.	**Is there a clear description of the programme or intervention or policy or reform on which the source material focusses?**	All source materials that describe an intervention or programme or policy.	• Are the rationale, goals or objectives of the programme or intervention or policy or reform described? • Is the content of the programme or intervention or policy described, including all of the important facets or elements? • Are the stakeholders or groups involved in delivering the programme or intervention or policy described, including their characteristics/background, skills or expertise, training and responsibilities? • Is the target/s of the programme or intervention or policy described? • Are the methods used to implement the programme or intervention or policy, including the mode of delivery (e.g. face‐to‐face, via the Internet) and any relevant training, described? • Are any materials used in the programme or intervention described? • Does the source material describe clearly any infrastructure and resources required for the programme or intervention or policy? • Does the source material describe when the programme or intervention or policy was started, when it finished, its intensity and whether there were any changes to the programme or intervention or policy over time? • Does the source material describe any mechanisms used to ensure that the programme or intervention or policy or reform was implemented as intended (e.g. supervision and support of personnel, training, implementation checks, incentives)?
4.	**Is there a clear description of the context/s to which the information described in the source material relates?**	All source materials	• Does the source material describe where the programme took place (e.g. country name(s), specific locations, urban/rural environments)? • Does the source material describe clearly the context for the material, including (where relevant): The setting (country, service, community) to which the description relates The system (e.g. health or welfare system), including the system level (e.g. frontline level) The historical, sociocultural, socioeconomic or ethical context The political, legal, governance, policy and/or practice context, including relevant key events or policy initiatives? • Does the source material describe clearly the stakeholders to which the description relates, including (where relevant): The target population(s) or group(s) for the programme or intervention or policy Implementing organisation(s) for the programme or intervention or policy Any other partners and stakeholders • Does the source material describe clearly how the different stakeholders were involved in the programme or intervention or policy or reform?
5.	**Is the information accurate?**	Source materials that include little or no empirical data	• Is there a clear description of whatever is the focus of the source material? • Does the information presented appear to be reasonably complete? • Does the source material describe any efforts to ensure that the information presented is complete and reliable?
6.	**Is the information accurate?**	Source materials that include empirical data	• Does the source material have clearly stated methods, including (where relevant) the type of empirical study conducted and when the programme or intervention or policy was evaluated? • Was the basis for selected cases or people or clusters appropriate for the purpose of the study? • Were the methods and tools for data collection appropriate for the purpose of the study? • Were the data collectors appropriately trained and supported in their tasks? • When were the data collected, and was the timespan of the study long enough to address the core issues fairly? • Was the quality of the data collected monitored and was the quality shown to be adequate? • Is the method of analysis reported clearly? Is the method of analysis appropriate for the purpose of the study? • Is there a clear description of the outcome/s measured? • Is the outcome measure reliable? • Were these outcomes measured appropriately? • Do these outcomes provide a reasonable assessment of the issue being considered? • Are the linkages between the data that were reported and any inferences made transparent?
7.	**Is the evidence representative? (With respect to population of interest, sampling frame etc.)**	All source materials	• If the evidence is drawn from a sample of the population of interest, is there a clear description of how the sampling was conducted? • Was the sampling approach appropriate (where applicable)? • If generalisations were made to wider population(s) or setting(s), is there a rationale for doing so and a description of how this was done?
8.	**Are any limitations of the information and/or methods discussed in the source material?**	All source materials	
9.	**Is evidence provided to support any findings or conclusions made?**	All source materials	• Are the findings or conclusions (where applicable) supported by evidence? • Are the findings or conclusions reasonable, in relation to the evidence presented?
10.	**Are relevant rights and ethics considerations described?**	Source materials that include empirical data	• The source material discusses relevant rights and ethics considerations • The source material indicates whether ethics approval was sought and obtained • The source material reports how consent to provide data or information was obtained

### Appendix 4. Table of review methods that were not implemented

Due to the limited number of studies included in the review of the primary objective, we were unable to conduct the following planned analysis.

**Table:**

**Method**	**Approach**
**Unit of analysis** **issues**	For clustered designs (such as cluster‐randomised trials), the reported results in included studies will often be on a level other than the level of allocation. If this is the case, we planned to perform an analysis adjusting for clustering, in order to avoid unit of analyses errors. If extracted results were not based on analyses adjusted for clustering, we planned to reanalyse the results (EPOC 2017c). If there was a unit of analysis error in the reported analysis for a study and there was insufficient information to reanalyse the results, a review author (LV) planned to contact the authors to request necessary data. We did not plan on reporting confidence intervals or P values for which there was a unit of analysis error, if these data were not available.
**Dealing with missing data**	We planned to contact investigators in order to verify key study characteristics and request missing outcome data where possible (e.g. when a study was identified as abstract only).
**Assessment of** **heterogeneity**	If we found a sufficient number of studies, we planned to conduct a meta‐analysis. We planned to use the I² statistic to measure heterogeneity among the trials in each analysis. If we identified substantial heterogeneity, we planned to explore it by prespecified subgroup analysis.
**Assessment of reporting biases**	We planned to contact study authors, asking them to provide missing outcome data. Where this was not possible, and the missing data were thought to introduce serious bias, we planned to explore the impact of including such studies in the overall assessment of results**.** If we were able to pool more than 10 trials, we planned to create and examine a funnel plot to explore possible publication biases, interpreting the results with caution (Sterne 2011).
**Data synthesis**	A common way that trialists indicate when they have skewed data is by reporting medians and interquartile ranges. If we encountered this, we proposed to note that the data were skewed and considered the implication of this. Where multiple trial arms were reported in a single trial, we planned to include only the relevant arms. If two comparisons (e.g. intervention A versus usual care and intervention B versus usual care) needed to be entered into the same meta‐analysis, we proposed to halve the control group to avoid double counting.
**Subgroup analysis and investigation of heterogeneity**	If meaningful, we planned to carry out the following subgroup analyses: by study setting (e.g. high‐income versus low‐ and middle‐income countries; urban versus rural); by whether there was an existing CIVIL REGISTRATION AND VITAL STATISTICS (paper‐based) system in place versus no CIVIL REGISTRATION AND VITAL STATISTICS system in place at all; by whether the notification was formal (i.e. for civil registration) versus informal (for purposes other than civil registration). We proposed to use the following outcomes in subgroup analysis. For birth notifications via mobile device: coverage (e.g. proportion) of births notified via mobile device; timeliness of birth notifications via mobile device (e.g. time between birth and birth notification via mobile device); timeliness of receipt of newborn or child health services (e.g. immunisations) in response to birth notifications via mobile device (i.e. time between birth and receipt of services). For death notifications via mobile device: coverage (e.g. proportion) of deaths notified via mobile device; timeliness of death notifications via mobile device (i.e. time between death and death notification via mobile device); timeliness of cause of death ascertainment, reporting to a disease surveillance system, or both, in response to death notifications via mobile device (i.e. time between death and cause of death ascertainment).
**Sensitivity analysis**	We planned to perform three sensitivity analyses to assess the robustness of our conclusions, and explore the impact on effect sizes. We proposed to restrict the analysis (i) to published studies, and (ii) to studies with a low risk of bias. For outcomes where acceptability or satisfaction was assessed quantitatively, we planned to (iii) exclude studies using unvalidated scales.
**Outcome data for** **secondary objectives**	For the **secondary objectives**, we planned to go through the included studies to assess whether any included robust outcome data (e.g. studies that reported results based on objective measures, from high quality, routine information systems). For studies that contained robust outcome data: we planned to extract relevant outcome data, if applicable; we planned to report the outcome data appropriately in the review.

### Appendix 5. GRADE‐CERQual evidence profiles

**Table:**

**Summary of review finding**		**Studies contributing to the review finding**	**Methodological limitations**	**Coherence**	**Adequacy**	**Relevance**	**Overall GRADE‐CERQual assessment of confidence in the evidence**	**Explanation for assessment**	
**A. Health system constraints in the implementation of birth and death notification via mobile devices**									
**A.1**	Geographic barriers hamper timeliness of birth and death notification conducted via mobile devices, as well as post‐notification services or processes (e.g. certification of birth or death).	ANISA 2016; MBRT 2016; MOVE‐IT 2013; mVRS 2017; Ngabo 2012; Pascoe 2012; Xeuatvongsa 2016	Serious concerns since 3 studies had significant limitations, 2 studies had minor limitations, and 2 studies had no or few limitations	Few or no concerns	Few or no concerns	Few or no concerns	**Moderate confidence**	Serious concerns related to methodological limitations. Few or no concerns related to coherence, relevance and adequacy	
**A.2**	Birth and death data collected using mobile devices can help health and civil registration systems identify problems and introduce appropriate quality improvements.	MBRT 2016; Moshabela 2015; MVH 2017; NIMDS 2019; RapidSMS 2012	Serious concerns	Few or no concerns	Serious concerns due to thinness of evidence	Few or no concerns	**Low confidence**	Serious concerns related to methodological limitations and adequacy. Few or no concerns with coherence and relevance	
**A.3**	Health workers who lack familiarity with, or prior experience in, using mobile technologies may need rigorous training as well as post‐training support.	Andreatta 2011; Gisore 2012; MBRL 2011; MBRT 2016; MOVE‐IT 2013; mSIMU 2017; mTika 2016; Ngabo 2012; NIMDS 2019; Van Dam 2015; Xeuatvongsa 2016; Yugi 2016	Moderate concerns since 2 studies had significant limitations, 6 studies had minor limitations, and 4 studies had no or few limitations	Few or no concerns	Few or no concerns	Few or no concerns	**Moderate confidence**	Moderate concerns related to methodological limitations. Few or no concerns related to coherence, relevance, and adequacy	
**A.4**	Local capacity to train future cadres of notifiers may be strengthened though 'train the trainer' approaches.	MBRL 2011; Ngabo 2012	Serious concerns since 1 study had significant limitations and 1 study had no or few limitations	Few or no concerns	Serious concerns due to thinness of evidence	Few or no concerns	**Low confidence**	Serious concerns related to methodological limitations and adequacy. Few or no concerns with coherence and relevance	
**A.5**	Mechanisms for continuous monitoring and supportive supervision are important for ensuring the quality and timeliness of birth and death data collected via mobile devices.	Andreatta 2011; ImTeCHO 2015; MOVE‐IT 2013; mTika 2016; Ngabo 2012; Pascoe 2012; Yugi 2016	Moderate concerns since 1 study had significant limitations, 4 studies had minor limitations, and 2 studies had no or few limitations	Few or no concerns	Moderate concerns due to thinness of evidence	Few or no concerns	**Moderate confidence**	Moderate concerns related to methodological limitations and adequacy. Few or no concerns with coherence and relevance	
**A.6**	Inadequate attention is paid to legal frameworks governing civil registration. These may need to be modified to allow notification via mobile device and the inclusion of new cadres of notifiers (low‐confidence finding).	eCRVS‐Mozambique 2017; MBRP 2015; mVRS 2017	Serious concerns since all 3 studies had significant limitations	Few or no concerns	Serious concerns due to thinness of evidence	Few or no concerns	**Low confidence**	Serious concerns related to methodological limitations and adequacy. Few or no concerns with coherence and relevance	
**A.7**	The availability of adequate human resources to conduct birth and death notification via mobile devices may be facilitated by hiring new cadres of notifiers or recruiting existing cadres of health workers to undertake notification.	Andreatta 2011; ANISA 2016; eCRVS‐Mozambique 2017; Gisore 2012; MBRL 2011; MBRT 2016; MOVE‐IT 2013; mVRS 2017; Pascoe 2012; Xeuatvongsa 2016; Yugi 2016	Serious concerns since 5 studies had serious limitations, 4 studies had minor limitations, and 2 studies had no or few limitations	Few or no concerns	Few or no concerns	Few or no concerns	**Moderate confidence**	Serious concerns related to methodological limitations. Few or no concerns with coherence, relevance, and adequacy	
**A.8**	Implementing birth and death notification via mobile devices may be influenced by underlying health and civil registration system infrastructure, resources, and processes.	ANISA 2016; Gisore 2012; MBRL 2011; Moshabela 2015; MOVE‐IT 2013; MVH 2017; mVRS 2017; Ngabo 2012	Serious concerns since 2 studies had significant limitations, 3 studies had minor limitations, and 3 studies had no or few limitations	Few or no concerns	Minor concerns due to thinness of evidence	Few or no concerns	**Low confidence**	Serious concerns related to methodological limitations. Minor concerns related to adequacy. Few or no concerns with coherence, and relevance	
**B. Factors related to individuals providing birth and death notification via mobile devices**									
**B.1**	Costs incurred by health workers sending notification using personal mobile phones may need to be reimbursed to facilitate sustained use of these technologies for notification.	Gisore 2012; mSIMU 2017; Ngabo 2012; Pascoe 2012; Xeuatvongsa 2016; Yugi 2016	Moderate concerns since 1 study had significant limitations, 2 studies had minor limitations, and 3 studies had no or few limitations	Few or no concerns	Moderate concerns due to thinness of evidence	Few or no concerns	**Moderate confidence**	Moderate concerns related to methodological limitations and adequacy. Few or no concerns related to coherence or relevance	
**B.2**	The use of mobile phones for notification is acceptable to health workers, and helps them to undertake their job responsibilities.	mSIMU 2017; Ngabo 2012; NIMDS 2019; Pascoe 2012; Van Dam 2015; Yugi 2016	Moderate concerns since 1 study had significant limitations, 3 studies had minor limitations, and 2 studies had no or few limitations	Few or no concerns	Moderate concerns due to thinness of evidence	Few or no concerns	**Moderate confidence**	Moderate concerns related to methodological limitations and adequacy. Few or no concerns related to coherence and relevance	
**B.3**	Health workers’ adoption of mobile birth and death notification strategies may be affected by competing priorities and the availability of adequate incentives.	MOVE‐IT 2013; mSIMU 2017; mTika 2016; MVH 2017	Minor concerns since 3 studies had minor limitations and 1 study had no or few limitations	Few or no concerns	Serious concerns due to thinness of evidence	Few or no concerns	**Moderate confidence**	Minor concerns related to methodological limitations. Serious concerns related to adequacy. Few or no concerns related to coherence and relevance	
**C. Factors related to families for whom birth and death is notified via mobile devices**									
**C.1**	For some families, costs may be a barrier to completing birth and death registration post‐notification.	MBRP 2015; MBRT 2016; MOVE‐IT 2013	Serious concerns since 2 studies had significant limitations and 1 study had minor limitations	Few or no concerns	Few or no concerns	Few or no concerns	**Low confidence**	Serious concerns related to methodological limitations and adequacy. Few or no concerns related to coherence, relevance, and adequacy	
**C.2**	There may be a need for targeted demand generation activities in communities with low awareness of the need of birth and death registration, alongside the use of mobile phones for birth and death notification.	MBRG 2014; MBRT 2016; MOVE‐IT 2013; mVRS 2017	Serious concerns since 3 studies had significant limitations, 1 study had minor limitations	Few or no concerns	Serious concerns due to thinness of evidence	Few or no concerns	**Low confidence**	Serious concerns related to methodological limitations. Moderate concerns related to adequacy. Few or no concerns related to coherence and relevance	
**C.3.**	Sociocultural norms may influence the timely identification of births and deaths, and should be taken into consideration when developing mobile phone interventions for notification.	ANISA 2016; MBRG 2014; MBRP 2015; MOVE‐IT 2013	Serious concerns since 2 studies had significant limitations, and 2 studies had minor limitations	Few or no concerns	Serious concerns due to thinness of evidence	Few or no concerns	**Low confidence**	Serious concerns related to methodological limitations and adequacy. Few or no concerns related to coherence and relevance	
**C.4**	Birth and death notification may increase access to these services for some families. However, they may also increase inequities in access related to low availability of supportive infrastructure (network coverage, roads, human resources), human factors (age, gender, literacy, poverty), and selective funding priorities of donors.	Andreatta 2011; Gisore 2012; MBRP 2015; MBRT 2016; MOVE‐IT 2013; mSIMU 2017; mTika 2016; mVRS 2017; Ngabo 2012; Xeuatvongsa 2016; Yugi 2016	Serious concerns since 3 studies had significant concerns, 5 studies had minor limitations, and 3 studies had no or few limitations	Few or no concerns	Few or no concerns	Few or no concerns	**Moderate confidence**	Serious concerns related to methodological limitations. Few or no concerns related to coherence, relevance, and adequacy	
**D. Factors related to government involvement in birth and death notification via mobile devices**									
**D.1**	Strong government commitment is a key factor in the successful implementation of birth and death notification via mobile devices.	eCRVS‐Mozambique 2017; MBRL 2011; MBRP 2015; MBRT 2016; mVRS 2017; Ngabo 2012; Yugi 2016	Serious concerns since 5 studies had significant limitations, 1 study had minor limitations, and 1 study had no or few limitations	Few or no concerns	Moderate concerns due to thinness of evidence	Few or no concerns	**Low confidence**	Serious concerns related to methodological limitations. Moderate concerns related to adequacy. Few or no concerns related to coherence or relevance	
**E. Factors related to technologies used for birth and death notification via mobile devices**									
**E.1**	Cost is an important consideration in the purchase, set‐up, and scaling up of mobile technologies needed for birth and death notification.	Gisore 2012; mTika 2016; mVRS 2017; Ngabo 2012; Pascoe 2012; Van Dam 2015; Xeuatvongsa 2016; Yugi 2016	Serious concerns since 2 studies had significant limitations, 3 studies had minor limitations, and 3 studies had no or few limitations	Few or no concerns	Moderate concerns due to thinness of evidence	Few or no concerns	**Low confidence**	Serious concerns related to methodological concerns. Moderate concerns related to adequacy. Few or no concerns related to coherence and relevance	
**E.2**	Challenges when notifying births and deaths via mobile devices include poor access to electricity and incompatibility with existing systems.	Gisore 2012; MBRL 2011; MBRP 2015; MBRT 2016; Ngabo 2012; Pascoe 2012	Serious concerns since 4 studies had significant limitations, and 2 studies had no or few limitations	Few or no concerns	Moderate concerns due to thinness of evidence	Few or no concerns	**Low confidence**	Serious concerns related to methodological concerns. Moderate concerns related to adequacy. Few or no concerns related to coherence and relevance	
**E.3**	The availability of network connectivity is a key factor in the successful implementation and scale‐up of birth and death notification via mobile devices.	ANISA 2016; MBRP 2015; MBRT 2016; MOVE‐IT 2013; mSIMU 2017; mVRS 2017; Ngabo 2012; NIMDS 2019; Pascoe 2012; Van Dam 2015; Yugi 2016	Serious concerns since 4 studies had significant limitations, 5 studies had minor limitations, and 2 studies had no or few limitations	Few or no concerns	Few or no concerns	Few or no concerns	**Moderate confidence**	Serious concerns related to methodological limitations. Few or no concerns with coherence, relevance, and adequacy	
**E.4**	Data security and encryption measures are needed to preserve confidentiality of birth and death information notified via mobile devices.	MBRT 2016; MVH 2017; Ngabo 2012; Van Dam 2015	Serious concerns since 1 study had significant limitations, 1 study had minor limitations and 1 study had no or few limitations	Few or no concerns	Serious concerns due to thinness of evidence	Few or no concerns	**Low confidence**	Serious concerns with methodological limitations and adequacy. Few or no concerns with coherence and relevance	

### Appendix 6. Questions that may help health system or programme managers when implementing or planning for birth or death notification via mobile device

1. Have you taken the needs and view of notifiers and community members into account when developing and planning the birth or death notification system?
2. Do notifiers have the legal authority to provide the services expected of them?
3. Will the planned notifiers have enough time within their current roles to deliver birth‐death notification services timeously?
4. Will families have reasonable access to notifiers and to post‐notification services?
5. Are there systems in place to analyse birth and death data to identify important health problems and trends?
6. Have the costs to the health system and to notifiers been included in the budget?
7. Have you assessed and taken into account the technological requirements for notifiers and for existing electronic health information systems?
8. Will the planned birth or death notification system reduce rather than increase inequities?
9. Is there a plan for addressing the training needs of notifiers?
10. Is there a plan for monitoring notifiers and providing supportive supervision?

## Characteristics of studies

### Characteristics of included studies [ordered by study ID]

**Table:** Andreatta 2011

***Study characteristics***		
Methods	Description of the implementation of a programme; service data reporting	
Participants	Birth attendants	
Interventions	Birth and death notification	
Outcomes	Outcomes related to the secondary objectives: Description of strategies used to implement birth and death notification via mobile devices Factors affecting the implementation of birth and death notifications via mobile device	
Context	Ghana (Sene district)	
Notes	Included in the review of secondary objectives only. Description of implementation factors was based on empirical data.

**Table:** ANISA 2016

***Study characteristics***		
Methods	Programmatic observations and lessons learned from the Aetiology of Neonatal Infection in South Asia (ANISA) study	
Participants	Families of pregnant women, Lady Health Workers (LHWs), Traditional Birth attendants (TBAs), or residents of the village with mobile phone access	
Interventions	Birth notification	
Outcomes	Outcomes related to the secondary objectives: Description of strategies used to implement birth and death notification via mobile devices Factors affecting the implementation of birth and death notifications via mobile device	
Context	Pakistan (Matiari district)	
Notes	Included in the review of secondary objectives only

**Table:** eCRVS‐Mozambique 2017

***Study characteristics***		
Methods	Description of the implementation of a programme	
Participants	Village chiefs	
Interventions	Birth and death notification	
Outcomes	Outcomes related to the secondary objectives: Description of strategies used to implement birth and death notification via mobile devices Factors affecting the implementation of birth and death notifications via mobile device	
Context	Mozambique	
Notes	Included in the review of secondary objectives only

**Table:** Gisore 2012

***Study characteristics***		
Methods	Uncontrolled before‐after evaluation study; description of the implementation of a programme conducted as part of the Global Network of Women's and Children's Health Birth Registry	
Participants	Village elders; registry administrator	
Interventions	Birth and death notification	
Outcomes	Outcomes related to the secondary objectives: Description of strategies used to implement birth and death notification via mobile devices Factors affecting the implementation of birth and death notifications via mobile device	
Context	Kenya (Western Province)	
Notes	Included in the review of secondary objectives only

**Table:** MBRG 2014

***Study characteristics***		
Methods	News article	
Participants	Community volunteers	
Interventions	Birth and death notification	
Outcomes	Outcomes related to the secondary objectives: Description of strategies used to implement birth and death notification via mobile devices Factors affecting the implementation of birth and death notifications via mobile device	
Context	Ghana	
Notes	Included in the review of secondary objectives only

**Table:** MBRL 2011

***Study characteristics***		
Methods	Description of the implementation of a programme	
Participants	Health workers	
Interventions	Birth notification	
Outcomes	Outcomes related to the secondary objectives: Description of strategies used to implement birth and death notification via mobile devices Factors affecting the implementation of birth and death notifications via mobile device	
Context	Liberia (Bomi county)	
Notes	Included in the review of secondary objectives only

**Table:** MBRP 2015

***Study characteristics***		
Methods	Description of the implementation of a programme	
Participants	Community 'gatekeepers' (marriage registrars, lady health workers, and Telenor (telecom) agents)	
Interventions	Birth notification	
Outcomes	Outcomes related to the secondary objectives: Description of strategies used to implement birth and death notification via mobile devices Factors affecting the implementation of birth and death notifications via mobile device	
Context	Pakistan (Panjab and Sindh provinces)	
Notes	Included in the review of secondary objectives only

**Table:** MBRT 2016

***Study characteristics***		
Methods	Description of the implementation of a programme	
Participants	Health workers in government clinics	
Interventions	Birth notification	
Outcomes	Outcomes related to the secondary objectives: Description of strategies used to implement birth and death notification via mobile devices Factors affecting the implementation of birth and death notifications via mobile device	
Context	Tanzania (Mbeya and Mwanza regions)	
Notes	Included in the review of secondary objectives only. Description of implementation factors was not based on empirical data.

**Table:** Moshabela 2015

***Study characteristics***		
Methods	Description of the implementation of a programme	
Participants	Community health workers	
Interventions	Birth and death notification	
Outcomes	Outcomes related to the secondary objectives: Description of strategies used to implement birth and death notification via mobile devices Factors affecting the implementation of birth and death notifications via mobile device	
Context	Senegal (Northwest)	
Notes	Included in the review of secondary objectives only

**Table:** MOVE‐IT 2013

***Study characteristics***		
Methods	Description of the implementation of a programme	
Participants	Village Executive Officers (VEOs)	
Interventions	Birth and death notification	
Outcomes	Outcomes related to the secondary objectives: Description of strategies used to implement birth and death notification via mobile devices Factors affecting the implementation of birth and death notifications via mobile device	
Context	Tanzania (Rufiji district)	
Notes	Included in the review of secondary objectives only

**Table:** mSIMU 2017

***Study characteristics***		
Methods	Description of intervention development and feasibility; cluster‐randomised trial for evaluation	
Participants	Village reporters working with the Health and Demographic Surveillance System (HDSS) programme	
Interventions	Birth notification	
Outcomes	Outcomes related to the secondary objectives: Description of strategies used to implement birth and death notification via mobile devices Factors affecting the implementation of birth and death notifications via mobile device	
Context	Kenya (Nyanza province)	
Notes	Included in the review of secondary objectives only

**Table:** mTika 2016

***Study characteristics***		
Methods	Controlled before‐after study; description of the implementation of an intervention	
Participants	Mothers	
Interventions	Birth notification	
Outcomes	Outcomes related to the secondary objectives: Description of strategies used to implement birth and death notification via mobile devices Factors affecting the implementation of birth and death notifications via mobile device	
Context	Bangladesh	
Notes	Included in the review of secondary objectives only. Some description of implementation factors based on qualitative interview data.

**Table:** MVH 2017

***Study characteristics***		
Methods	Description of the implementation of a programme	
Participants	Staff at all Turkey health cluster organisations (internal partners)	
Interventions	Death notification	
Outcomes	Outcomes related to the secondary objectives: Description of strategies used to implement birth and death notification via mobile devices Factors affecting the implementation of birth and death notifications via mobile device	
Context	Syria, Turkey	
Notes	Included in the review of secondary objectives only

**Table:** mVRS 2017

***Study characteristics***		
Methods	Economic evaluation	
Participants	Village chiefs	
Interventions	Birth notification	
Outcomes	Outcomes related to the secondary objectives: Description of strategies used to implement birth and death notification via mobile devices Factors affecting the implementation of birth and death notifications via mobile device	
Context	Uganda	
Notes	Included in the review of secondary objectives only

**Table:** Ngabo 2012

***Study characteristics***		
Methods	Description of the implementation of a programme	
Participants	Community health workers	
Interventions	Birth and death notification	
Outcomes	Outcomes related to the secondary objectives: Description of strategies used to implement birth and death notification via mobile devices Factors affecting the implementation of birth and death notifications via mobile device	
Context	Rwanda (Northern province)	
Notes	Included in the review of secondary objectives only

**Table:** NIMDS 2019

***Study characteristics***		
Methods	Description of the implementation of a programme	
Participants	Lady Health Supervisors (LHSs)	
Interventions	Death notification	
Outcomes	Outcomes related to the secondary objectives: Description of strategies used to implement birth and death notification via mobile devices Factors affecting the implementation of birth and death notifications via mobile device	
Context	Pakistan (Punjab district)	
Notes	Included in the review of secondary objectives only

**Table:** Pascoe 2012

***Study characteristics***		
Methods	Description of the implementation of a programme	
Participants	Health workers	
Interventions	Death notification	
Outcomes	Outcomes related to the secondary objectives: Description of strategies used to implement birth and death notification via mobile devices Factors affecting the implementation of birth and death notifications via mobile device	
Context	Tanzania (Pwani region)	
Notes	Included in the review of secondary objectives only

**Table:** RapidSMS 2012

***Study characteristics***		
Methods	Description of the implementation of a programme	
Participants	Registrars	
Interventions	Birth notification	
Outcomes	Outcomes related to the secondary objectives: Description of strategies used to implement birth and death notification via mobile devices Factors affecting the implementation of birth and death notifications via mobile device	
Context	Nigeria	
Notes	Included in the review of secondary objectives. Description of implementation factors not based on empirical data

**Table:** Van Dam 2015

***Study characteristics***		
Methods	Feasibility and usability evaluation; description of the implementation of a programme	
Participants	Health workers	
Interventions	Death notification	
Outcomes	Outcomes related to the secondary objectives: Description of strategies used to implement birth and death notification via mobile devices Factors affecting the implementation of birth and death notifications via mobile device	
Context	Zambia (Lusaka)	
Notes	Included in the review of secondary objectives only

**Table:** Xeuatvongsa 2016

***Study characteristics***		
Methods	Controlled before‐after study; description of the implementation of a programme	
Participants	Village Health Workers (VHVs); Health Care Workers (HCWs)	
Interventions	Birth notification	
Outcomes	Outcomes related to the primary objectives: Coverage of births notified via mobile device Timeliness of birth notification via mobile device Coverage of newborn or child health services in response to birth notification via mobile device Timeliness of receipt of newborn or child health services in response to birth notification via mobile device Outcomes related to the secondary objectives: Description of strategies used to implement birth and death notification via mobile devices Factors affecting the implementation of birth and death notifications via mobile device	
Context	Lao People’s Democratic Republic (Luang and Xayabuly provinces)	
Notes	Included in the review of primary and secondary objectives

**Table:** Yugi 2016

***Study characteristics***		
Methods	Description of the implementation of a programme; routine surveillance data	
Participants	Health facility staff, County Health Department staff	
Interventions	Death notification	
Outcomes	Outcomes related to the secondary objectives: Description of strategies used to implement birth and death notification via mobile devices Factors affecting the implementation of birth and death notifications via mobile device	
Context	South Sudan (Eastern Equatoria state)	
Notes	Included in the review of secondary objectives only	

^ANISA: Aetiology of Neonatal Infection in South Asia study ASHA: Accredited Social Health Activist HCW: Health Care Worker HDSS:^^Health and Demographic Surveillance Systems LHS: Lady Health Supervisor LHW: Lady Health Worker pg: Page TBA: Traditional Birth Attendant VEO: Village Executive Officer VHV: Village Health Volunteer^

### Characteristics of excluded studies [ordered by study ID]

**Table:**

Study	Reason for exclusion
Adler‐Milstein 2014	Did not report on births or deaths
Alehagen 2012	Did not report on births or deaths
Amoah 2016	Did not report on births or deaths
Arnold 2013	Did not report on births or deaths
Benski 2017	Did not report on births or deaths
Boak 2008	Not conducted via mobile devices
Bogaerts 2017	Did not report on births or deaths
Bogebjerg 2012	Used existing data source
Boggon 2013	Did not report on births or deaths
Callahan 2011	Review article
Centers 2012	Did not report on births or deaths
Chalo 2005	Did not report on births or deaths
Chen 2014	Did not report on births or deaths
Chiba 2012	Not conducted via mobile device
Clark 2015	Used existing data source
Clayman 2006	Used existing data source
Clements 2015	Did not report on births or deaths
Coleman 2017	Did not report on births or deaths
Danovaro‐Holliday 2014	Review article
DeJoy 2014	Not conducted via mobile device
De Savigny 2017	Did not report on births or deaths
Devasenapathy 2015	Did not report on births or deaths
Elliott 2010	Used existing data source
Fan 2010	Not conducted via mobile device
Fazen 2013	Did not report on births or deaths
Fisher 2017	Not conducted via mobile device
Froen 2016	Review article
Ghebrehewet 2003	Did not report on births or deaths
Godefay 2016	Did not report centralised system or focal individuals for birth or death notification
Gorman 2015	Did not report on births or deaths
Griebenow 2013	Did not report on births or deaths
Gyllstrom 2002	Used existing data source
Ha 2013	Did not report on births or deaths
Harron 2016	Used existing data source
Haskew 2015	Not conducted via mobile device
Hassan 2017	Did not report centralised system or focal individuals for birth or death notification
Hobgood 2005	Did not report on births or deaths
Huq 2014	Not conducted via mobile device
ImTeCHO 2015	Notification component not described
Irgens 2002	Review article
Jimoh 2012	Did not report on births or deaths
Jo 2014	Did not report on births or deaths
Joukes 2015	Did not report on births or deaths
Kabadi 2015	Not conducted via mobile device
Khodadost 2015	Not conducted via mobile device
Lee 2016	Review article
Lee 2016a	Did not report on births or deaths
Li 2013	Did not report on births or deaths
Lima 2014	Used existing data source
Lingaas 2004	Did not report on births or deaths
Little 2013	Did not report on births or deaths
Lopez 2015	Review article
Lucas 2010	Used existing data source
Maslowsky 2016	Did not report on births or deaths
Mathews 2006	Used existing data source
Matthew 2016	Review article
McKenna 2002	Did not report on births or deaths
Melo 2004	Used existing data source
Michael 2013	Did not report on births or deaths
Moreno‐Iribas 2013	Used existing data source
Mumtaz 2014	Did not report on births or deaths
Munro 2014	Did not report on births or deaths
Nakamura 2013	Did not report on births or deaths
Nau 2015	Did not report on births or deaths
Ngoma 2015	Did not report on births or deaths
Nie 2016	Did not report on births or deaths
Okaro 2001	Used existing data source
Oyeyemi 2014	Did not report on births or deaths
Patterson 2004	Did not report on births or deaths
Pearson 2009	Review article
Pector 2004	Did not report on births or deaths
Pell 2016	Did not report on births or deaths
Prieto 2017	Did not report on births or deaths
Rothstein 2016	Did not report on births or deaths
Rowe 2016	Did not report on births or deaths
Rowley 2008	Did not report on births or deaths
Schlumberger 2015	Did not report on births or deaths
Shaheen 2014	Did not report on births or deaths
Singogo 2013	Not conducted via mobile device
Steinke 2004	Did not report on births or deaths
Suresh 2005	Review article
Taggart 2012	Did not report on births or deaths
Tesfaye 2014	Used existing data source
Tesfaye 2017	Unclear if conducted via mobile device
Tourassi 2016	Used existing data source
Tran 2013	Not conducted via mobile device
Van Lier 2014	Did not report on births or deaths
Wang 2006	Used existing data source
Wang 2007	Used existing data source
Wintermeyer‐Pingel 2013	Did not report centralised system or focal individuals for birth or death notification
Xu 2016	Did not report on births or deaths
Yu 2015	Used existing data source
Yu 2016	Did not report on births or deaths
Zhang 2016	Did not report on births or deaths
Zoban 2010	Did not report on births or deaths
Zotti 2002	Not conducted via mobile device
Zurcher 2017	Review article

### Characteristics of studies awaiting classification [ordered by study ID]

**Table:** Haar 2018

Methods	Field surveillance programme
Participants	Syrian American Medical Society (SAMS)
Interventions	Mobile data collection questionnaire using Magpi platform to collect data on incidents of attacks on healthcare
Outcomes	Number of deaths
Context	Syria (Gaziantep)
Notes	To be assessed for inclusion in the review of secondary objectives

SAMS: Syrian American Medical Society

### Characteristics of ongoing studies [ordered by study ID]

**Table:** Helleringer 2018

Study name	Measuring HIV‐related mortality during surveys in Africa
Methods	Randomised trial
Participants	Individuals aged 15‐59 years old who have ever resided in the Karonga Health and Demographic Surveillance Systems (KHDSS) area and whose deceased sibling death was either HIV‐related or not related to HIV
Interventions	Mortality questionnaire administered using Open Data Kit platform on mobile devices
Outcomes	Sensitivity, defined as the proportion of HIV positive deaths according to KHDSS that were correctly reported as such by survey respondents Specificity, defined as the proportion of HIV‐negative deaths according to KHDSS that were correctly reported as such by survey respondents
Starting date	June 1, 2017
Contact information	Stephane Helleringer Johns Hopkins Bloomberg School of Public Health, MD, USA Email: sheller7@jhu.edu
Context	Malawai (Karonga Health and Demographic Surveillance Systems (KHDSS) area)
Notes	Author not contacted yet; trial end date listed as May 31, 2019

**Table:** Ostermann 2019

Study name	mPARIS: Mobile phone‐based (mHealth) reminders and incentives for improving vaccination timeliness
Methods	Quasi‐randomised trials
Participants	Pregnant women; mother‐baby dyads; family members
Interventions	Text message reminders and mobile phone vouchers for timely vaccinations; pregnant woman or family can notify study about birth using USSD
Outcomes	Number of vaccination visits within 4 weeks of scheduled visit dates
Starting date	August 15, 2017
Contact information	Lavanya Vasudevan Duke University, NC, USA Email: lavanya.vasudevan@duke.edu
Context	Tanzania (Mtwara region)
Notes	Outcome data not yet available

**Table:** Uddin 2017

Study name	Mobile phone‐based technology to improve health, population and nutrition services
Methods	Controlled before‐after study
Participants	Healthcare providers; pregnant women; family members
Interventions	Digital tracking system; pregnant women or family members can notify study of a birth using SMS
Outcomes	Antenatal care (ANC), delivery care, postnatal care (PNC), neonatal care, expanded programme on immunisation (EPI) coverage, and contraceptive prevalence rate (CPR)
Starting date	2017
Contact information	Jasim Uddin International Centre for Diarrhoeal Disease Research, Dhaka, Bangladesh Email: jasim@icddrb.org
Context	Bangladesh (Rajshahu and Chittagong divisions)
Notes	Emailed study author on Nov 14, 2017. Outcome data not yet available

^ANC: Antenatal Care CPR: Contraceptive Prevalence Rate EPI: Expanded Programme on Immunisation HIV: Human Immununodeficiency Virus KHDSS:^^Karonga^^ Health and Demographic Surveillance Systems mPARIS: Mobile phone‐based (mHealth) reminders and incentive system PNC: Postnatal Care SMS: Short Message Service USDD:Unstructured Supplementary Service Data^

## Differences between protocol and review

Review methods that were described in the protocol but not implemented are listed in Appendix 4.
